# Real-world effectiveness of biological therapy in patients with rheumatoid arthritis: Systematic review and meta-analysis

**DOI:** 10.3389/fphar.2022.927179

**Published:** 2022-08-11

**Authors:** Caroline Tianeze de Castro, Mariana Jorge de Queiroz, Flavia Caixeta Albuquerque, Celmário Castro Brandão, Leticia Farias Gerlack, Daniella Cristina Rodrigues Pereira, Sandra Castro Barros, Wenderson Walla Andrade, Ediane de Assis Bastos, Jessé de Nobrega Batista Azevedo, Roberto Carreiro, Mauricio Lima Barreto, Djanilson Barbosa dos Santos

**Affiliations:** ^1^ Institute of Collective Health, Federal University of Bahia, Salvador, Brazil; ^2^ Department of Pharmaceutical Assistance and Strategic Inputs, Ministry of Health, Rio de Janeiro, Brazil; ^3^ Oswaldo Cruz Foundation (FIOCRUZ), Rio de Janeiro, Brazil; ^4^ Center of Data and Knowledge Integration for Health (CIDACS), Gonçalo Moniz Institute, Oswaldo Cruz Foundation (FIOCRUZ), Salvador, Brazil; ^5^ Center for Health Sciences, Federal University of Recôncavo da Bahia, Santo Antônio de Jesus, Brazil

**Keywords:** rheumatoid arthritis, biological therapy, meta-analysis, effectiveness, administrative health databases

## Abstract

**Background:** The treatment of rheumatoid arthritis (RA), a chronic systemic inflammatory autoimmune disease, is based on disease-modifying anti-rheumatic drugs (DMARDs). Typically, it starts with conventional synthetic DMARDs (csDMARDs), and depending on the patient’s response to the treatment and the adverse events experienced, biological DMARDs (bDMARDs) are initiated. bDMARDs are more specific to inflammatory factors than csDMARDs and more efficient in inducing remission and low disease activity. Thus, this study aimed to assess the effectiveness of biological therapy in patients with rheumatoid arthritis in administrative health databases.

**Methods:** PubMed, Embase, Lilacs, Ovid, Scopus, and Web of Science databases were searched from inception to 21 October 2021, to identify observational studies that evaluated the effectiveness of biological therapy in patients with rheumatoid arthritis using administrative databases and real-world data. The methodological quality was assessed by the methodological index for non-randomized studies (MINORS). A fixed or random-effects model estimated risk ratios with 95% confidence intervals. The analysis was divided into four groups: tumor necrosis factor inhibitors (TNFi) versus non-TNFi; TNFi versus TNFi (adalimumab, etanercept, and golimumab versus infliximab); bDMARDs versus Janus kinase inhibitors (JAKi); and bDMARDs monotherapy versus combination therapy (bDMARDs and MTX).

**Results:** Twenty-one records were eligible for inclusion in this systematic review and meta-analysis; seven population-based cohorts, eight prospective, and six retrospective cohort studies. Overall, 182,098 rheumatoid arthritis patients were evaluated. In the meta-analysis, lower effectiveness was observed among TNFi users than in non-TNFi (RR: 0.88; 95% CI: 0.81–0.95; *p* < 0.01; I^2^ = 94.0%) and bDMARDs than in JAKi (RR: 0.86; 95% CI: 0.79–0.94; *p* < 0.01; I^2^ = 93.0%). Higher effectiveness among adalimumab, etanercept, and golimumab than in infliximab (RR: 1.19; 95% CI: 1.05–1.36; *p* < 0.01; I^2^ = 96.0%) was found. No significant differences in the effectiveness of bDMARD monotherapy compared to combination therapy (RR: 0.83; 95% CI: 0.68–1.00; *p* < 0.01; I^2^ = 81.0%) was observed. E-value analysis indicated that the estimates were not robust against unmeasured confounding.

**Conclusion:** According to the available real-world data, our results suggest that biological therapy effectively treats patients with rheumatoid arthritis, indicating higher effectiveness with non-TNFi and JAKi than with TNFi.

**Systematic Review Registration:** https://www.crd.york.ac.uk/prospero/display_record.php?ID#CRD42020190838, identifier CRD42020190838.

## 1 Introduction

Rheumatoid arthritis (RA) is a chronic systemic inflammatory autoimmune disease that affects the synovial fluid of joints, tendons, and some extra-articular sites, leading to deformity and destruction of joints by bone erosion and cartilage destruction (Guo et al., 2018; Lin et al., 2020). It is estimated that 0.4–1.3% of the world population is affected by the disease, which is two to four times more frequent in women. The age at onset is commonly situated around 30 years, with a peak in the fifth decade of life (Amaya-Amaya et al., 2013; Lin et al., 2020).

Treatment for RA aims to reduce disease activity state, through clinical remission or at least achievement of low disease activity, especially for patients with previous treatment failure. RA treatment is based on disease-modifying anti-rheumatic drugs (DMARDs), typically starting with conventional synthetic DMARDs (csDMARDs) as methotrexate (MTX), hydroxychloroquine, and sulfasalazine, and depending on the patient’s response to the treatment and the adverse events experienced, biological DMARDs (bDMARDs) are initiated to reduce RA symptoms, slow disease progression, and improve physical function (Smolen et al., 2020).

Several bDMARDs have recently emerged in RA management, including TNF-α inhibitors (TNFi) as adalimumab, etanercept, and infliximab; IL-6 receptor antibody, such as tocilizumab; and JAK inhibitors (JAKi) as tofacitinib (Guo et al., 2018; Smolen et al., 2020). However, despite the wide range of biological medicines available, their real-world effectiveness is still under discussion.

There is uncertainty about the effectiveness of TNFi with the first and subsequent uses. In many observational studies, slightly better retention rates and effectiveness have been reported for etanercept than for adalimumab and infliximab, but there is some uncertainty about whether this superiority reflects channeling bias or an actual difference (Lee et al., 2008; Hetland et al., 2010). Consequently, direct evidence of the effectiveness of TNFi is needed to inform clinical and drug reimbursement decision-makers.

Previous systematic reviews and meta-analyses of randomized clinical trials (RCTs) have shown improvement in the remission rates of RA patients with first-line TNFi versus placebo (with or without MTX) (Gulácsi et al., 2019), and better response rates in subjects are exposed to tocilizumab and sarilumab than to adalimumab (Sung and Lee, 2021). Nonetheless, a systematic review and network meta-analysis of 28 RCTs compared the efficacy of csDMARDs, TNFi, non-TNFi, and JAKi with abatacept and found no significant differences between these drugs (Paul et al., 2020).

Although RCTs evaluate the efficacy of treatments in selected groups of patients defined by strict inclusion criteria, the value of these trials in predicting therapeutic effectiveness in “real-world” patients is limited. This systematic review and meta-analysis were designed to complement the knowledge obtained in RCTs and observational studies with primary data by evaluating the real-world effectiveness of TNFi in patients with RA in observational studies with administrative health databases.

Therefore, this systematic review and meta-analysis aimed to assess the real-world effectiveness of biological therapy in patients with rheumatoid arthritis in observational studies with administrative health databases.

## 2 Methods

This systematic review and meta-analysis followed the Preferred Reporting Items for Systematic Reviews and Meta-analyses (PRISMA) statement (Page et al., 2021). The protocol for this systematic review was registered in the International Prospective Register of Systematic Review (PROSPERO) database before starting the literature search (CRD42020190838).

### 2.1 Eligibility criteria and outcome measures

The PECOS structure was adopted to define the eligibility criteria. Therefore, the population of interest (P) was patients with rheumatoid arthritis, the exposure (E) was the use of biological drugs (adalimumab, certolizumab pegol, etanercept, golimumab, infliximab, abatacept, rituximab, and tocilizumab), the comparator (C) was patients with rheumatoid arthritis unexposed to biological drugs or exposed to different drug classes, the outcome of interest (O) was therapeutic effectiveness, and the study design (S) was observational studies.

Effectiveness was the main outcome of interest for this study. Effectiveness was considered as remission or improvement of disease activity, measured by the Disease Activity Score 28 (DAS28), European Alliance of Associations for Rheumatology (EULAR), Clinical Disease Activity Index (CDAI), or The Simplified Disease Activity Index (SDAI); improvement in functional capacity, measured by the Health Assessment Questionnaire (HAQ); persistence in therapy; or other measures adopted by the studies.

Other outcomes associated with effectiveness explored in this systematic review and meta-analysis were: the reduction of clinical disease activity assessed by ACR70 (70% reduction criteria of the American College of Rheumatology), ACR50 (50% reduction criteria of the American College of Rheumatology), drug withdrawal, and maintenance of remission after withdrawal of the drug.

Observational studies (prospective cohort, retrospective cohort, and case-control) with administrative databases and real-world data were eligible for inclusion. No language or date restrictions were applied. Clinical trials, review articles, case reports, case series, and animal studies were excluded.

### 2.2 Search strategy

Searches were conducted in Embase, Lilacs, Ovid, PubMed, Scopus, and Web of Science databases to identify studies that assessed the effectiveness of biological therapy in patients with rheumatoid arthritis from inception to 21 October 2021. In addition, grey literature sources were searched (Catálogo de Teses e Dissertações da CAPES and specialized journals) to identify any studies that were not indexed in the databases but might be relevant for inclusion in the present systematic review. Search process details are presented in Supplementary Table S1.

### 2.3 Study selection and data extraction

Articles’ titles and abstracts were independently evaluated by two reviewers (CCB and LG) for potentially relevant articles using Rayyan (Ouzzani et al., 2016). The studies that met the inclusion criteria in the first stage had their eligibility confirmed by reading the full article. The qualitative and quantitative synthesis included those that met all the inclusion criteria. A third reviewer (DBS) was consulted when the reviewers disagreed on whether an article should be included.

Two reviewers independently extracted the included studies’ details (MJQ and FCA). The extracted data include authors, journal, publication year, country, sample size, effectiveness outcomes, statistical analysis method (including statistical tests and measure of association with confidence intervals), and adjustment variables (confounders).

### 2.4 Methodological quality assessment

Two reviewers (CTC and MJQ) assessed the methodological quality of the included studies using the methodological index for non-randomized studies (MINORS) (Slim et al., 2003), a validated index to assess the quality of observational studies. This tool contains 12 questions, with a global ideal score for comparative studies of 24 points. The quality assessment of the included studies was measured as follows: 0 to 6 points, very low quality; 7 to 12 points, low quality; 13 to 18 points, moderate quality; and 19 to 24 points, high quality (Pithon et al., 2019).

### 2.5 Statistical analysis

Data were extracted from eligible studies and arranged in 2 × 2 tables. Risk ratios (RRs) and 95% confidence intervals (95% CI) were calculated by the fixed or the random-effects model, depending on the heterogeneity between the studies. The I^2^ statistic and Cochran’s Q test were adopted to evaluate heterogeneity and consistency (Higgins, 2003). The random-effects model was applied when heterogeneity was verified (I^2^ > 50%; *p* < 0.05). The analysis was divided into four groups: TNFi versus non-TNFi; TNFi versus TNFi (adalimumab, etanercept, and golimumab versus infliximab); bDMARDs versus JAKi; and bDMARD monotherapy versus combination therapy (bDMARDs and MTX). A subgroup analysis by effectiveness measure was conducted. Publication bias was assessed by visual inspection of the funnel plot and statistically using Egger’s tests. A minimum of ten studies were considered to elaborate on this graph and judge the risk of bias associated with missing data (Page et al., 2020). Analyses were carried out with R version 4.1.2 and the “meta” package version 4.13-0 (Balduzzi et al., 2019).

### 2.6 Sensitivity analysis

Sensitivity analyses were performed, stratifying the analysis by prior use of bDMARDs and no prior use of bDMARDs since bDMARD-naïve patients have a greater response to bDMARDs than those with previous exposure to bDMARDs (Wakabayashi et al., 2011; Mori et al., 2018).

Additionally, an evaluation of how sensitive the estimates from each study were to the effects of unmeasured confounders was performed through the E-value. This measure represents an unmeasured confounder’s strength to make a reported exposure-outcome association statistically non-significant (Mathur and VanderWeele, 2020). Thus, the size of unobserved confounding able to nullify the mean risk ratio was quantified, and the unmeasured confounding strengths sufficient to allow 10% of studies with true RR above or below a threshold to remain statistically significant were calculated for each one of the four groups analyzed.

## 3 Results

### 3.1 Selected studies

**FIGURE 1:**
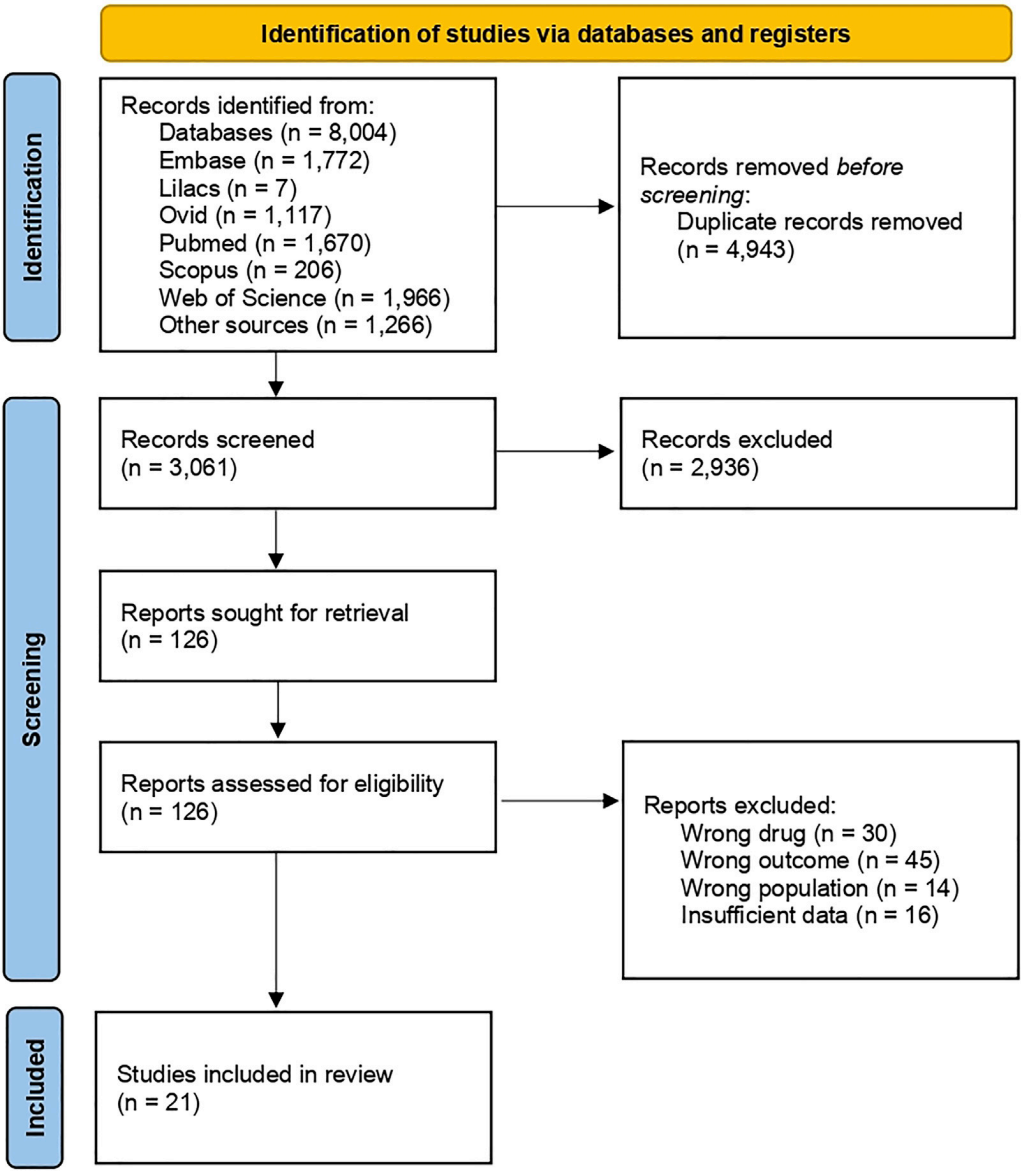
Flow chart of search results.

The initial search returned 8,004 records, of which 4,943 were duplicates. After screening titles and abstracts, 126 studies were analyzed regarding inclusion criteria, and 105 were excluded. Afterward, references to the included studies were manually searched to detect relevant articles, but none was identified. Therefore, articles were excluded from analyzing the wrong drug, outcome, and population and from having insufficient data (Figure 1).

### 3.2 Study characteristics

Twenty-one records were eligible for inclusion in this systematic review; seven population-based cohorts (Østergaard et al., 2007; Chatzidionysiou et al., 2015; Neovius et al., 2015; Acurcio et al., 2016; Kihara et al., 2017; Choi et al., 2021; Li et al., 2021), eight prospective (Harrold et al., 2015; Lauper et al., 2018; Ebina et al., 2020a, 2020b; Rahman et al., 2020; Curtis et al., 2021; Pappas et al., 2021a, 2021b), and six retrospective cohort studies (Curtis et al., 2015; Yun et al., 2015; Silvagni et al., 2018; Bird et al., 2020; Gharaibeh et al., 2020; Youssef et al., 2020), which are published from 2007 to 2021 (Supplementary Table S2).

Overall, 182,098 rheumatoid arthritis patients were evaluated; the majority were women (67–88%), and the mean age ranged between 48 and 70 years (Supplementary Table S2). Disease duration was between 4 and 13 years. Most studies included patients with moderate to high disease activity, indicating severe rheumatoid arthritis and poor prognosis (Table 1).

**TABLE 1:** Characteristics of the included studies.

Study	Year	Country	Patients	Mean disease duration (years)	Mean disease activity	Prior use of bDMARDs	Current use of steroids	Outcome
Acurcio	2016	Brazil	76,351	NR	NR	NR	NR	Medication persistence in the 1st and 2nd year
Bird	2020	Australia	1,950	8.9–10.0	NR	No	NR	Medication persistence and improvement and remission in DAS28, CDAI, and SDAI
Chatzidionysio	2014	Sweden	7,052	8.4–9.9	DAS28: 4.7–5.1	Yes	Yes	Improvement, remission, and change in DAS28 and therapy discontinuation in 6 months
Choi	2021	South Korea	8,018	NR	NR	Yes	NR	Drug failure and medication persistence
Curtis	2015	United States of America	5,474	NR	NR	NR	Yes	Effectiveness (high adherence, no increase in biologic dose, no biologic switch, no new DMARD, no new/increased oral glucocorticoid, and ≤1 glucocorticoid injection)
Curtis	2021	United States of America	1,270	7.3–9.2	CDAI: 31.5–33.2	Yes	NR	Change in CDAI at months 6 and 12
Ebina	2020 (a)	Japan	3,897	4.7–9.2	DAS28-ESR: 4.1–4.6	Yes	Yes	Treatment discontinuation
Ebina	2020 (b)	Japan	221	7.8–11.6	DAS28-CRP: 3.2–3.9	Yes	Yes	Treatment discontinuation
Gharaibeh	2020	United States of America	14,775	NR	NR	No	Yes	Nonadherence, increased index medication dose, addition of a conventional DMARD, switch of biologic medications, addition of glucocorticoid or increased glucocorticoid dose, and receipt of ≥2 intra-articular injections in 1 year
Harrold	2015	United States of America	1,398	11.5–13.4	CDAI: 21.4–22.9	Yes	Yes	Responsiveness to medication treatment based on improvement in CDAI, modified ACR20 (mACR20), modified ACR50 (mACR50), and modified ACR70 (mACR70) responses at 6th and 12th month
Kihara	2017	United Kingdom	2,636	4.0–5.0	DAS28: 6.0–6.2	Yes	Yes	Change in DAS28, EULAR response, DAS28 remission, change in HAQ score, and proportion of patients who achieved the minimal clinically important difference in HAQ at the 6th month
Lauper	2018	Czech Republic, Finland, Italy, Norway, Portugal, Romania, Russia, Slovenia, Spain, and Switzerland	8,308	7.9–10.2	DAS28: 4.0–4.6	Yes	Yes	Medication persistence, change in CDAI, and DAS28-ESR in 1 year
Li	2021	Taiwan	8,663	NR	NR	No	NR	Treatment discontinuation and switching
Neovius	2015	Sweden	9,139	12–13	DAS28: 5.1–5.2	No	NR	Therapy discontinuation due to any cause (except for pregnancy and remission) and remission in 5 years
Østergaard	2007	Denmark	300	NR	DAS28-CRP: 5.9	No	NR	DAS28 and EULAR response rates at week 26 and 52
Pappas	2021 (a)	United States of America	617	8.8	CDAI: 3.5–3.7	No	Yes	Medication persistence, discontinuation, and switching
Pappas	2021 (b)	United States of America	4,816	7.1–8.6	CDAI: 20.4	No	Yes	Improvement in CDAI and DAS28, remission based on CDAI and DAS28, and change in CDAI, HAQ, and EQ-5D
Rahman	2020	Canada	1,577	6.5–9.8	DAS28-CRP: 4.1–5.3	Yes	Yes	Medication discontinuation, improvement in DAS28 and HAQ-DI, SDAI remission, and low disease activity
Silvagni	2018	Italy	4,478	5.0	NR	No	Yes	Medication persistence
Youssef	2020	Australia	6,914	10.0	NR	Yes	Yes	Medication persistence
Yun	2015	United States of America	14,244	NR	NR	No	Yes	No switch to a different biologic, high adherence to the index drug, no addition of a new non-biologic DMARD, no biologic dose increase compared with starting, no initiation of glucocorticoids/no increase in dose, and no more than one joint injection on unique days after 3 months of new treatments

ACR, American College of Rheumatology; CDAI, Clinical Disease Activity Index; DAS28, Disease Activity Score-28; DAS28-CRP, DAS-28C-reactive protein; DAS28-ESR, DAS-28 erythrocyte sedimentation rate; DMARD, disease-modifying anti-rheumatic drug; EULAR, European Alliance of Associations for Rheumatology; HAQ, Health Assessment Questionnaire; HAQ-DI, HAQ Disability Index; NR, not reported; SDAI, Simplified Disease Activity Index.

Ten studies compromised RA patients in second-line therapy (Chatzidionysiou et al., 2015; Harrold et al., 2015; Kihara et al., 2017; Lauper et al., 2018; Ebina et al., 2020a, 2020b; Rahman et al., 2020; Youssef et al., 2020; Choi et al., 2021; Curtis et al., 2021), nine in first-line therapy (Østergaard et al., 2007; Neovius et al., 2015; Yun et al., 2015; Silvagni et al., 2018; Bird et al., 2020; Gharaibeh et al., 2020; Li et al., 2021; Pappas et al., 2021b, 2021a), and two did not report this information (Curtis et al., 2015; Acurcio et al., 2016) (Table 1).

Studies evaluated second-line therapy with tocilizumab versus TNFi (monotherapy or combination therapy with csDMARDs) after the use of at least one bDMARD (Lauper et al., 2018); second-line treatment with bDMARDs and tsDMARD after the use of other bDMARDs and tsDMARD (Youssef et al., 2020); and second and third-line bDMARDs and tsDMARD (Choi et al., 2021). One research included patients with previous therapy with bDMARDs and concurrent DMARDs (Curtis et al., 2021). Another study indicated a proportion of biologic-experienced RA patients of 6.3–19.7% (Rahman et al., 2020).

Five reports evaluated therapy switching, of which one analyzed switching from first TNFi to second TNFi (Chatzidionysiou et al., 2015); one from TNFi to abatacept and other TNFi (Harrold et al., 2015); one from any bDMARD to tocilizumab (Kihara et al., 2017); one from any bDMARD to another bDMARD or tofacitinib (Ebina et al., 2020a); and one from tocilizumab or abatacept after failure to bDMARDs or JAKi (Ebina et al., 2020b). The first four studies did not report the duration of the first therapy. The last one observed mean therapy duration between 16.4 and 26.7 months for tocilizumab and 10.9 and 11.0 months for abatacept.

Furthermore, fourteen records described the proportion of RA patients in current use of steroids (Chatzidionysiou et al., 2015; Curtis et al., 2015; Yun et al., 2015; Harrold et al., 2015; Kihara et al., 2017; Silvagni et al., 2018; Lauper et al., 2018; Ebina et al., 2020a, 2020b; Rahman et al., 2020; Youssef et al., 2020; Gharaibeh et al., 2020; Pappas et al., 2021a, 2021b), which ranged from 9.9 to 78.0%; however, none of these studies did a separate analysis for patients who are currently exposed to steroids and unexposed to steroids.

The 21 studies investigated nine different biological drugs, among them TNFi (etanercept, infliximab, adalimumab, certolizumab pegol, golimumab, and tocilizumab), non-TNFi (rituximab and abatacept), and JAKi (tofacitinib). Additionally, three studies compared bDMARD monotherapy and combination therapy (bDMARDs and MTX).

Regarding the outcomes, most articles analyzed medication persistence, remission, and improvement in disease activity. The studies’ remission and disease activity measures encompassed DAS28, EULAR, CDAI, SDAI, and HAQ.

### 3.3 Quality of the included studies

According to the MINORS, twenty studies were classified as high quality (Chatzidionysiou et al., 2015; Curtis et al., 2015, 2021; Harrold et al., 2015; Neovius et al., 2015; Yun et al., 2015; Acurcio et al., 2016; Kihara et al., 2017; Lauper et al., 2018; Silvagni et al., 2018; Bird et al., 2020; Ebina et al., 2020a, 2020b; Gharaibeh et al., 2020; Rahman et al., 2020; Youssef et al., 2020; Choi et al., 2021; Li et al., 2021; Pappas et al., 2021a, 2021b) and one as moderate quality (Østergaard et al., 2007). Overall, studies scored between 14 and 24 points (Supplementary Table S3).

### 3.4 Meta-analysis

#### 3.4.1 TNFi versus non-TNFi

**FIGURE 2:**
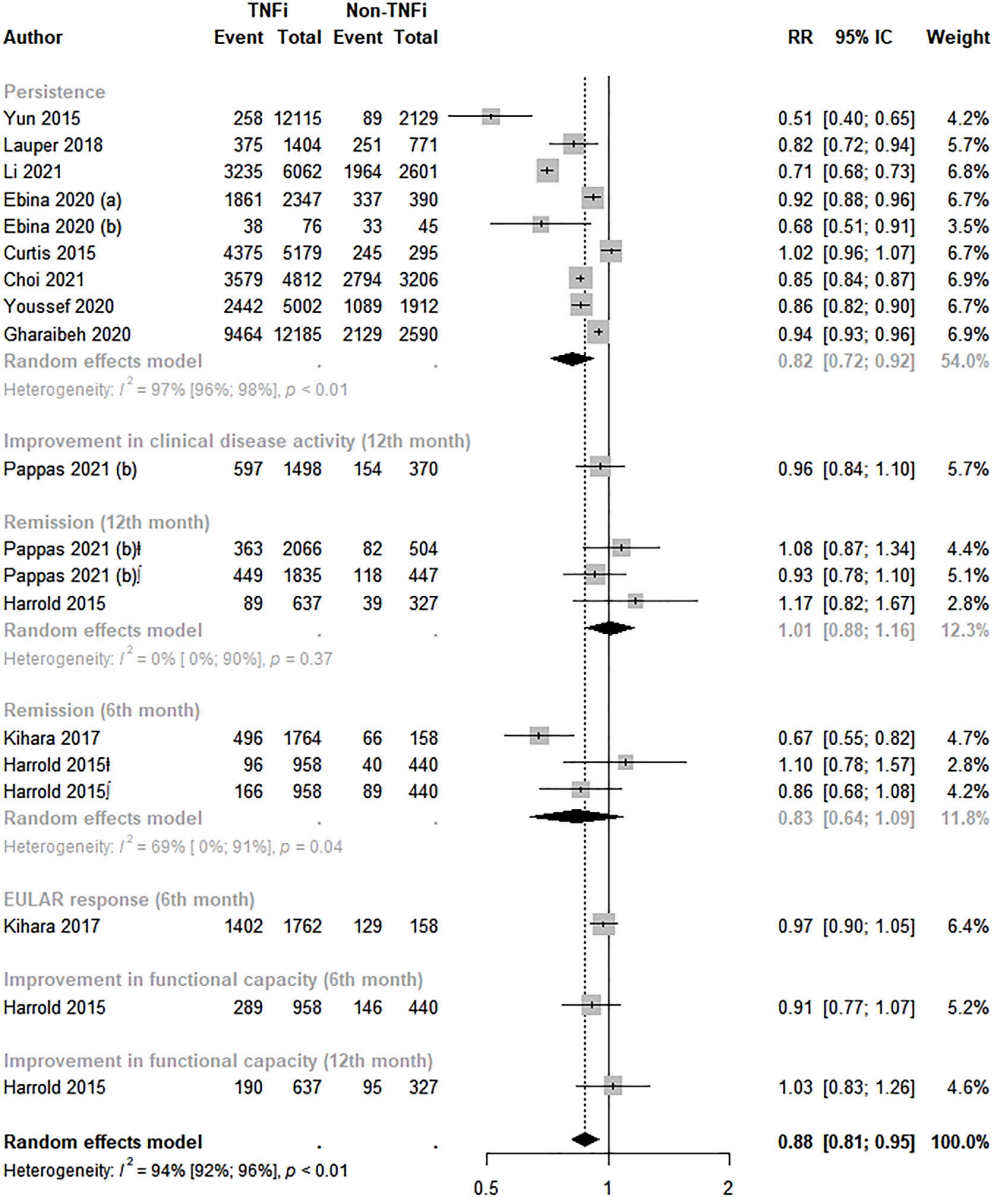
Effectiveness of TNF inhibitors compared to non-TNF inhibitors. TNFi, TNF inhibitors; non-TNFi, non-TNF inhibitors; ⱡ, remission based in CDAI; ∫, remission based in DAS28.

Twelve studies assessed the effectiveness between TNFi and non-TNFi (Curtis et al., 2015; Harrold et al., 2015; Yun et al., 2015; Kihara et al., 2017; Lauper et al., 2018; Ebina et al., 2020a, 2020b; Gharaibeh et al., 2020; Youssef et al., 2020; Choi et al., 2021; Li et al., 2021; Pappas et al., 2021b). A statistically significant lower effectiveness was observed among TNFi users than in non-TNFi users (RR: 0.88; 95% CI: 0.81–0.95; *p* < 0.01; I^2^ = 94.0%). The analysis by effectiveness measure revealed lower therapy persistence (RR: 0.82; 95% CI: 0.72–0.92) with TNFi than with non-TNFi drugs (Figure 2). Visual inspection of the funnel plot did not suggest asymmetry (Supplementary Figure S1), and Egger’s test did not indicate publication bias (intercept = −0.01, *p* = 0.99).

#### 3.4.2 Adalimumab, etanercept, and golimumab versus infliximab

**FIGURE 3:**
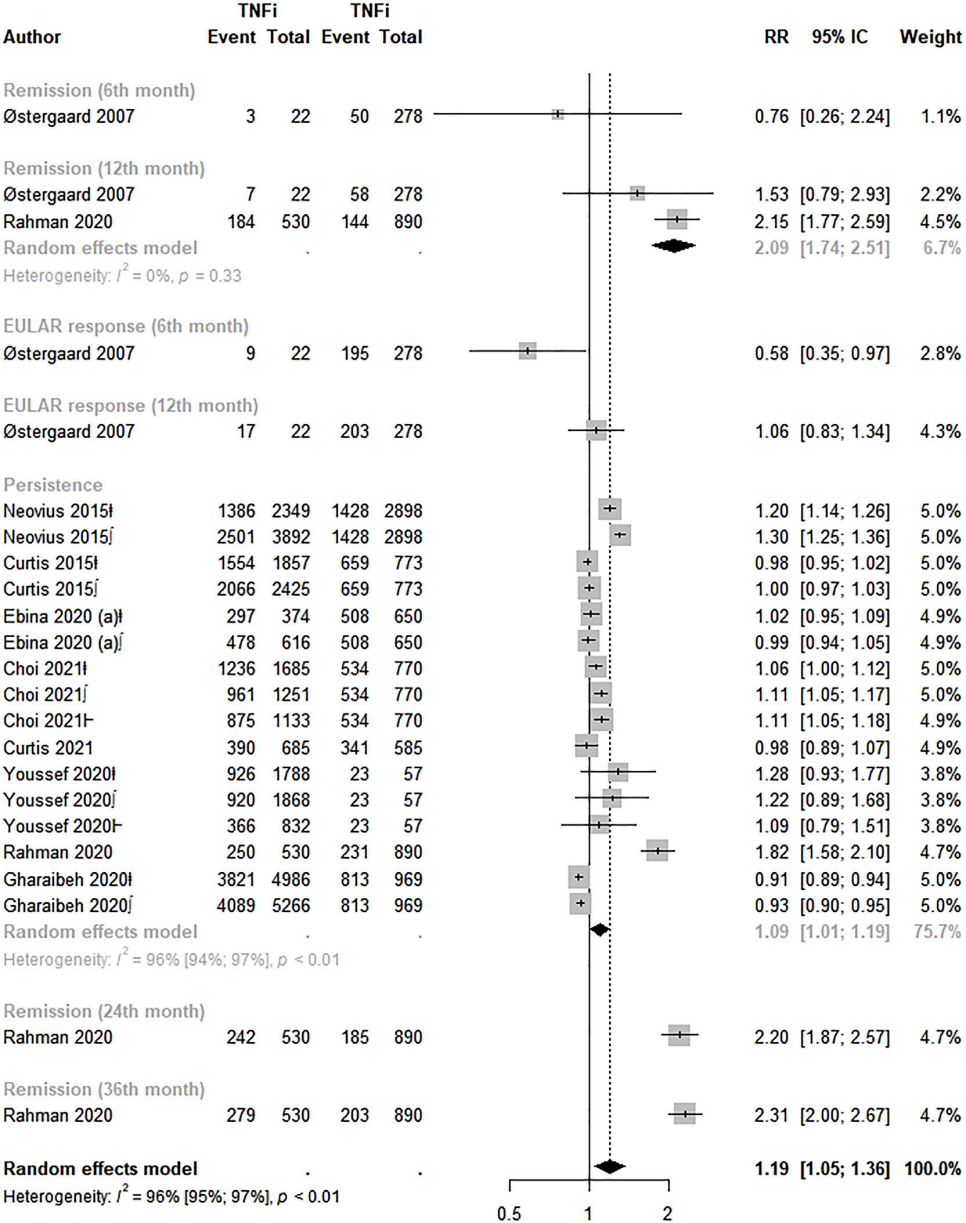
Effectiveness of Adalimumab, Etanercept and Golimumab compared to Infliximab. TNFi, TNF inhibitors; ⱡ, adalimumab; ∫, etanercept; Ⱶ, golimumab.

Ten studies evaluated the effectiveness of adalimumab, etanercept, and golimumab versus infliximab (Østergaard et al., 2007; Curtis et al., 2015, 2021; Neovius et al., 2015; Ebina et al., 2020a, 2020b; Gharaibeh et al., 2020; Rahman et al., 2020; Youssef et al., 2020; Choi et al., 2021). Overall, adalimumab, etanercept, and golimumab were 19.0% more effective for rheumatoid arthritis than infliximab (RR: 1.19; 95% CI: 1.05–1.36; *p* < 0.01; I^2^ = 96.0%). Higher therapy persistence (RR: 1.09; 95% CI: 1.01–1.19) and remission in the 12th month (RR: 2.09; 95% CI: 1.74–2.51) were pointed out in the analysis by effectiveness measure (Figure 3). Visual inspection of the funnel plot indicated asymmetry, suggesting publication bias (Supplementary Figure S2). Egger’s test indicated publication bias (intercept = 3.97, *p* = 0.02).

The analysis by drug showed a significant higher effectiveness of golimumab (RR: 1.57; 95% CI: 1.19–2.08; *p* < 0.01; I^2^ = 97.0%) over infliximab. However, the subgroup analysis by effectiveness measure did not reveal statistically significant results for adalimumab, etanercept, or golimumab over infliximab (Supplementary Figure S3).

#### 3.4.3 bDMARDs versus JAKi

**FIGURE 4:**
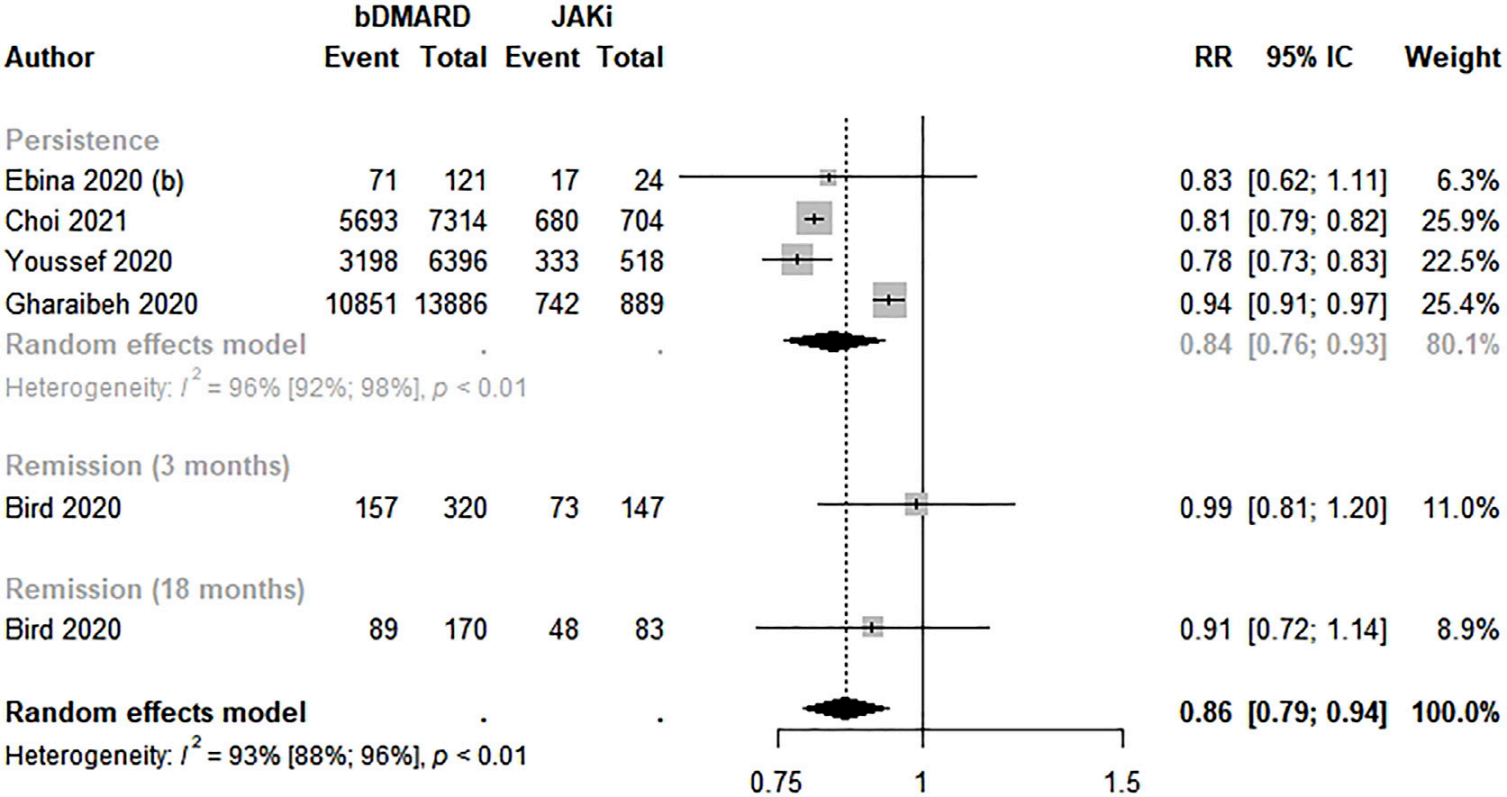
Effectiveness of biological disease-modifying anti-rheumatic drugs compared to Janus kinase inhibitors. bDMARD: biological disease-modifying anti-rheumatic drug; JAKi: Janus kinase inhibitors.

Five studies estimated the effectiveness of bDMARDs compared to JAKi (Bird et al., 2020; Ebina et al., 2020b; Gharaibeh et al., 2020; Youssef et al., 2020; Choi et al., 2021). bDMARDs were 14.0% less effective for rheumatoid arthritis than JAKi (RR: 0.86; 95% CI: 0.79–0.94; *p* < 0.01; I^2^ = 93.0%). Regarding the analysis by effectiveness measure, a lower persistence in bDMARD therapy was observed (RR 0.84; 95% CI 0.76–0.93) (Figure 4).

#### 3.4.4 bDMARD monotherapy versus combination therapy

**FIGURE 5:**
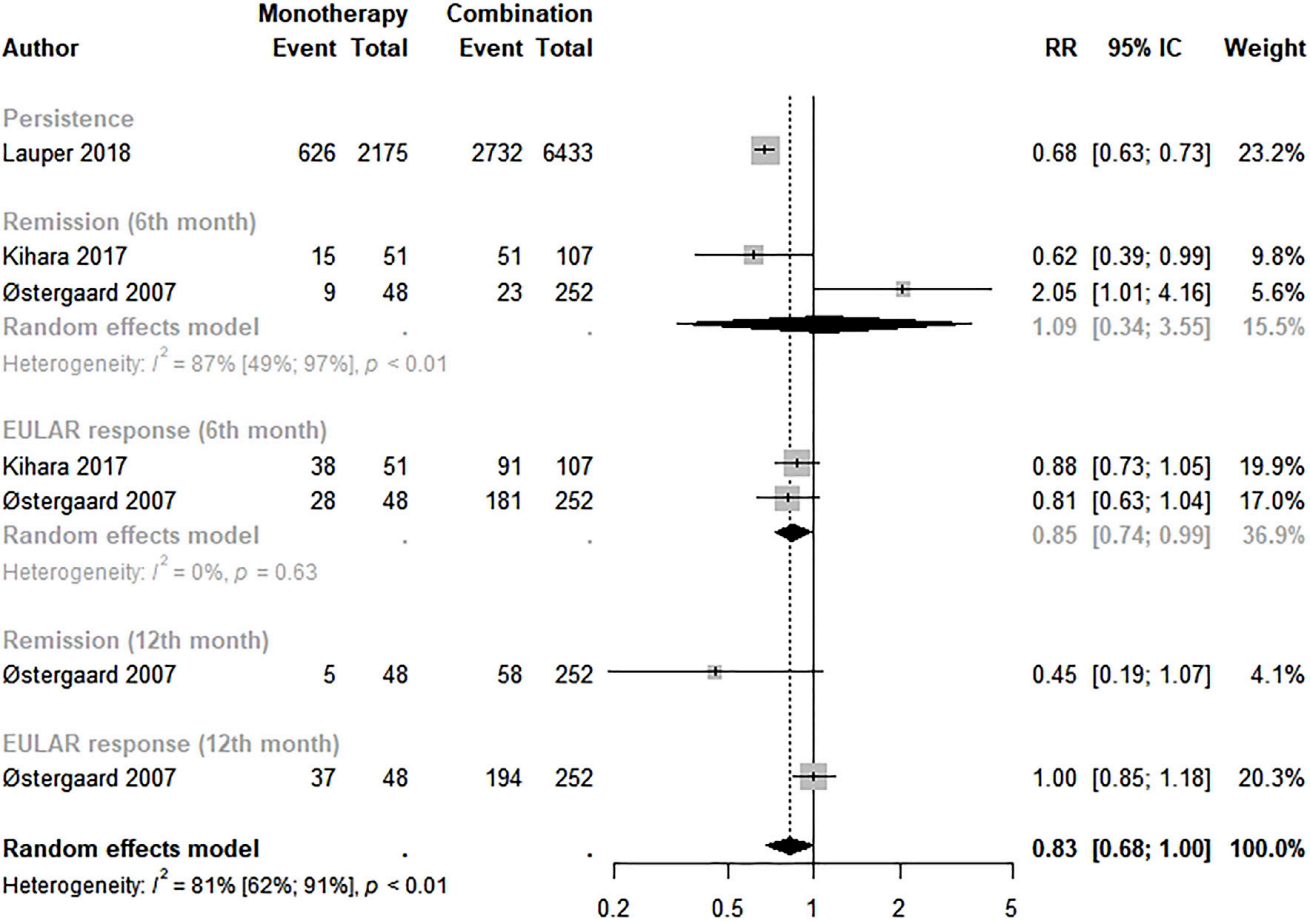
Effectiveness of biological disease-modifying anti-rheumatic drugs monotherapy compared to combination therapy. Monotherapy: biological disease-modifying anti-rheumatic drugs monotherapy; combination: biological disease-modifying anti-rheumatic drugs + methotrexate.

The effectiveness between bDMARD monotherapy and combination therapy was evaluated by three studies (Østergaard et al., 2007; Kihara et al., 2017; Lauper et al., 2018). The meta-analysis revealed a lower effectiveness of bDMARD monotherapy than of combination therapy with borderline statistical significance (RR: 0.83; 95% CI: 0.68–1.00; *p* < 0.01; I^2^ = 81.0%). However, a lower EULAR response in the 6th month with statistical significance was observed in bDMARD monotherapy (RR: 0.85; 95% CI: 0.74–0.99) (Figure 5).

### 3.5 Sensitivity analysis

In analyses by prior use of bDMARDs, a statistically significant lower effectiveness was observed among TNFi users than in non-TNFi users who had never been exposed to biological therapy (RR: 0.86; 95% CI: 0.78–0.95; *p* < 0.01; I^2^ = 96.0%), while non-significant differences were observed among biologic-experienced patients (Supplementary Figure S4).

In contrast, a 50.0% higher effectiveness was presented by biologic-experienced subjects exposed to adalimumab, etanercept, and golimumab than to infliximab (RR: 1.50; 95% CI: 1.15–1.95; *p* < 0.01; I^2^ = 96.0%). Regarding biologic-naïve patients, there were no significant differences between the drugs (RR: 1.05; 95% CI: 1.00–1.11; *p* < 0.01; I^2^ = 94.0%) (Supplementary Figure S5).

In the sensitivity analysis of bDMARDs compared to JAKi, bDMARDs had lower effectiveness than JAKi in biologic-naïve patients (RR: 0.86; 95% CI: 0.79–0.95; *p* < 0.01; I^2^ = 95.0%), and non-statistical significance was found among patients with prior use of biologics (Supplementary Figure S6).

Non-significant differences on effectiveness were observed in sensitivity analysis of bDMARD monotherapy compared to combination therapy among patients who had previously been exposed to biologic drugs (RR: 0.87; 95% CI: 0.66–1.14; *p* < 0.01; I^2^ = 94.0%) and those who had never been exposed to biologic drugs (RR: 0.89; 95% CI: 0.76–1.04; *p* < 0.01; I^2^ = 85.0%) (Supplementary Figure S7).

The sensitivity analysis for unmeasured confounding showed that an unobserved confounder needed to be associated with both TNFi use and effectiveness with a risk ratio of at least 1.65 (95% CI: 1.00–2.82) to reduce to less than 10% the percentage of meaningfully strong true causal effects. For adalimumab, etanercept, and golimumab and the outcome, a risk ratio of at least 1.97 (95% CI: 1.00–3.52) would be necessary to reduce to less than 10% the percentage of meaningfully strong true causal effects, while for bDMARDs and bDMARD monotherapy, the necessary risk ratios should be 1.61 (95% CI: 1.00–2.85) and 2.06 (95% CI: 1.00–4.08), respectively.

## 4 Discussion

This systematic review and meta-analysis provide a quantitative estimate of the real-world effectiveness of different biological therapies in patients with rheumatoid arthritis in studies using administrative health databases. Real-world effectiveness data provide valuable evidence to support the efficacy findings from randomized controlled trials (RCTs) (Blonde et al., 2018) once trial patients may not represent the real-world RA population.

Overall, this meta-analysis showed statistically significant differences in effectiveness between the biological medicines analyzed. For example, TNFi showed less effectiveness in RA patients than non-TNFi drugs, as well as bDMARDs compared to JAKi, and bDMARD monotherapy compared to combination therapy. In contrast, golimumab showed higher effectiveness than infliximab. However, it is important to highlight the low number of studies included in some analyses.

These findings are similar to the results of efficacy from previous RCTs. The ADACT and AMPLE trials compared the efficacy of tocilizumab versus adalimumab and abatacept versus adalimumab, respectively, and indicated greater effectiveness of non-TNFi over the TNFi analyzed (Gabay et al., 2013; Weinblatt et al., 2013). Regarding JAKi, RCT findings are controversial, pointing to the greater effectiveness of baricitinib over adalimumab (Keystone et al., 2017; Taylor et al., 2017) and lower effectiveness of tofacitinib than of adalimumab (Fleischmann et al., 2017).

The development of drugs to target TNF-α has been one of the most impressive advances in treating inflammatory diseases in the past decade. However, some patients do not tolerate or respond adequately to available TNFi. In these cases, other biologically derived drugs with different action mechanisms may be used, such as abatacept, which is a T-cell co-stimulation inhibitor, and JAKi, which are oral drugs counteracting the activation of cytosolic enzymes presiding over many biologic functions (JAKs) (Smolen et al., 2009; Angelini et al., 2020).

TNF-α is an important cytokine that mediates inflammation and bone degradation in RA through local inflammation and pannus formation, eventually leading to further cartilage erosion and bone destruction. The introduction of TNFi has revolutionized RA treatment options, resulting in the development of further biologic DMARDs (Ma and Xu, 2013). TNFi drugs act by reducing TNF-α levels in RA, restoring the balance in the cytokine system. Many TNFi drugs are available nowadays, including infliximab, adalimumab, etanercept, and golimumab. The first TNFi drug for RA was infliximab, a chimeric human-murine monoclonal antibody that binds with high affinity to soluble and transmembrane forms of TNF-α but not to lymphotoxin. Since the advent of infliximab, genetically engineered molecules employing a slightly different compositional and pharmacodynamic approach have been marketed (Pelechas et al., 2019).

Unlike the present results, where significant effectiveness of adalimumab over infliximab was not observed, ATTEST and AMPLE trials found higher efficacy of adalimumab than infliximab, with a statistically significant odds ratio of ACR20 (OR: 1.73; 95% CI: 1.04–2.87), ACR50 (OR: 1.49; 95% CI: 1.02–2.19), and low disease activity (DAS28) (OR: 2.12; 95% CI: 1.19–3.78) were observed among patients treated with adalimumab (Christensen et al., 2013). However, results from these RCTs reflect a limited population of RA patients, leading to limitations related to small sample size and exclusion criteria that limit generalizability to real-world subjects. So, these differences highlight real-world studies’ importance in investigating drug effects in clinical practice since the effectiveness of drug therapy depends on factors such as adherence to the medication and the outcomes associated with the drug use in different patient populations.

Although significantly higher effectiveness of etanercept over infliximab was not found in the meta-analysis, a retrospective cohort study with data from the CORRONA registry pointed out that patients on etanercept monotherapy experience greater therapy persistence in the 6th and 12th month and are less likely to reintroduce a csDMARD than patients on other TNFi monotherapies. The authors stated that the development of neutralizing anti-drug antibodies to TNFi other than etanercept might contribute to these findings (Pappas et al., 2021a).

Similar to the findings of this systematic review and meta-analysis, a systematic review of sixteen RCTs compared the efficacy of TNFi using Bayesian mixed treatment comparison models and found greater efficacy of golimumab than infliximab by the Health Assessment Questionnaire (HAQ) score (Schmitz et al., 2012). Golimumab is a human anti-TNF-α monoclonal antibody generated and matured in an *in vivo* system, with high affinity and specificity for human TNF-α, and effectively neutralizes TNF-α bioactivity (Ma and Xu, 2013). Furthermore, this biological drug presents low levels of immunogenicity and a more attractive dosage scheme (every 4 weeks) (Pelechas et al., 2019), which may influence its greater effectiveness than infliximab.

According to previous studies, using combination therapy (bDMARDs and MTX) contributes to a higher persistence of biological therapy in RA patients (Lauper et al., 2018). Similarly, patients treated with higher MTX doses tend to persist in treatment for a longer time (Soliman et al., 2011; Aaltonen et al., 2017). However, in the meta-analysis comparing bDMARD monotherapy to combination therapy, a borderline statistically significant lower effectiveness was found among patients treated exclusively with bDMARDs. This finding may be related to the evaluation of different biological medicines by each study, such as tocilizumab or TNFi (Lauper et al., 2018), tocilizumab (Kihara et al., 2017), and infliximab and etanercept (Østergaard et al., 2007).

Furthermore, the sensitivity analysis revealed lower effectiveness of TNFi versus non-TNFi and bDMARDs versus JAKi in biologic-naïve patients, indicating a possible benefit from non-TNFi and JAKi pharmacotherapy in these subjects. Regarding biologic-experienced subjects, higher effectiveness was observed with adalimumab, etanercept, and golimumab than with infliximab. Given the present findings, adalimumab, etanercept, and golimumab may be effective treatment options for patients with inadequate response to infliximab.

Most of the included studies evaluated as effectiveness measure therapy persistence, remission, and improvement in disease activity. Persistence in therapy is an excellent indirect and composite measure of effectiveness, safety, and tolerability, reflecting the long-term impact on the course of the disease (Silvagni et al., 2018). Twelve studies evaluated the therapy persistence in this systematic review (Curtis et al., 2015; Neovius et al., 2015; Yun et al., 2015; Lauper et al., 2018; Ebina et al., 2020a, 2020b; Gharaibeh et al., 2020; Rahman et al., 2020; Youssef et al., 2020; Choi et al., 2021; Curtis et al., 2021; Li et al., 2021). In addition, the majority of the studies that evaluated the therapy persistence of TNFi in comparison to non-TNFi found significant differences among the therapies, favoring non-TNFi over TNFi (Curtis et al., 2015; Yun et al., 2015; Lauper et al., 2018; Ebina et al., 2020a; Youssef et al., 2020; Choi et al., 2021; Li et al., 2021). The same pattern was observed among articles that assessed persistence among bDMARD and JAKi, showing lower persistence in bDMARD RA patients (Gharaibeh et al., 2020; Youssef et al., 2020; Choi et al., 2021). In contrast, only two articles found significant differences between adalimumab, etanercept, and golimumab versus infliximab, pointing to a higher persistence among RA patients exposed to adalimumab and etanercept than infliximab (Neovius et al., 2015; Rahman et al., 2020).

According to Yun et al. (2014), one in every three patients interrupts their treatments with the first bDMARD in the first year of use due to lack of efficacy and/or adverse events. Nonetheless, treating autoimmune diseases that cause systemic inflammation is vital since there is evidence that the persistence of systemic inflammation leads to a higher risk of death (Listing et al., 2015). Furthermore, RA patients present a higher risk of death due to cardiovascular events when compared to the general population (Zhang et al., 2016).

A critical treatment goal in managing RA patients is the achievement of clinical remission (Ajeganova and Huizinga, 2017). However, only six studies used this outcome as an effectiveness measure (Østergaard et al., 2007; Harrold et al., 2015; Kihara et al., 2017; Bird et al., 2020; Rahman et al., 2020; Pappas et al., 2021b). Furthermore, only one of the included studies observed significant differences among the biological therapies evaluated in clinical remission (Rahman et al., 2020). The prospective cohort used data from the Biologic Treatment Registry Across Canada (BioTRAC) between 2002 and 2017 and evaluated the effectiveness of golimumab and infliximab. The authors observed higher SDAI clinical remission at 12, 24, and 36 months in patients treated with golimumab (34.7, 47.5, and 52.7%, respectively) than in those treated with infliximab (of 16.2, 20.8, and 22.8%, respectively) (Rahman et al., 2020).

The expressive variation in the remission and disease activity measures adopted by the studies included in the present systematic review and meta-analysis, encompassing DAS28, EULAR, CDAI, SDAI, and HAQ, must be highlighted. A treat-to-target strategy is recommended in RA, and for this purpose, regular RA disease activity assessments must be made during routine care. Many RA disease activity measures are available that incorporate data gathered from a combination of sources, including patient-reported measures, provider assessments, laboratory values, and/or imaging modalities; nevertheless, these measures may vary in performance and feasibility (England et al., 2019). Considering these, the American College of Rheumatology (ACR) and European League Against Rheumatism (EULAR) recommend a variety of RA disease activity measures, such as CDAI, DAS28-ESR/CRP, and SDAI, for regular use (England et al., 2019; Smolen et al., 2020).

It is important to emphasize that because of the different immune-modulatory properties of specific drugs and drug classes, biological therapy may be related to several potential adverse events, such as hospitalized infection, solid cancers and lymphoma, cardiovascular diseases, and mortality (Yun et al., 2016). Therefore, the pharmacotherapy selection must consider not only the medicine’s efficacy but also its associated risk.

RA treatment has progressively improved over the last decades due to the contribution of biological therapies and treat-to-target strategies, which aim at the achievement of clinical remission by slowing or stopping the progression of joint destruction and deformity. This process improved therapeutic results and quality of life and reduced patient morbidity and mortality (Bullock et al., 2018; Ho et al., 2019). Furthermore, therapy choice depends on disease severity, the patient’s clinical response, and previously experienced side effects. Although biological medicines improve the likelihood of reaching the treatment target in many RA patients, they are costly, limiting their widespread use and contributing to the inequity of access across countries. Thus, they should be used in an evidence-based manner that accounts for availability and affordability within the local healthcare system (Ho et al., 2019; Smolen et al., 2020).

### 4.1 Strengths and limitations

This systematic review and meta-analysis present strengths and limitations. This is a comprehensive assessment of the evidence, incorporating all available published studies on the real-world effectiveness of biological therapies in patients with rheumatoid arthritis. Strengths also encompass studies with administrative health databases as inclusion criteria, random-effects meta-analysis to deal with the heterogeneity, and the conduction of sensitivity analysis stratified by prior use of bDMARDs and no prior use of bDMARDs.

A significant limitation is the possibility of findings by chance in the meta-analyses comparing bDMARDS versus JAKi and bDMARD monotherapy versus combination therapy due to the low number of studies included. Meta-analyses of small numbers of studies have limitations that can impact their findings, although they present valid results (Herbison et al., 2011). Also, it was not possible to analyze bDMARDs compared to csDMARDs since only one of the included studies evaluated this (Acurcio et al., 2016).

Also, it was not possible to perform sensitivity analyses by the duration of previous drugs because the included studies did not have this information and by RA patients currently exposed to steroids versus those unexposed to these medicines since none of the included studies reported patients unexposed to steroids.

Another limitation is the high heterogeneity between studies, which persisted after subgroup and sensitivity analyses. This could be justified by several factors such as differences in measures of effectiveness adopted, differences in RA severity and prognosis, and differences in some population characteristics.

The publication bias found in studies that evaluated TNFi compared to TNFi (infliximab) is probably associated with the eligibility criteria adopted, including only observational studies with administrative databases, usually resulting in more extensive studies.

Moreover, raw data were used to perform meta-analyses instead of adjusted measures, considering the variety of association measures and the several combinations of covariates submitted to the adjustment procedures by the studies. So, the type of analysis performed cannot control confounders such as age, gender, ethnicity, education, work, type of health insurance, body mass index (BMI), smoking, comorbidities, and use of drugs that can influence drugs’ effectiveness, such as steroids and NSAIDs.

Although real-world data may not be as rigorous as RCT data because of the string inclusion criteria, data collection, and quality control, it may lead to a better understanding of the effectiveness of biological therapy in a more complex and heterogeneous RA population, which is more representative of clinical practice.

## Data Availability

The original contributions presented in the study are included in the article/Supplementary Material; further inquiries can be directed to the corresponding author.
