# Supplementary material for: Real-world effectiveness of biological therapy in patients with rheumatoid arthritis: Systematic review and meta-analysis
Source: Front Pharmacol. 2022 Aug 11;13:927179. doi: 10.3389/fphar.2022.927179 (PMC9402894; doi:10.3389/fphar.2022.927179)
Supplement: Supplementary file 1 [file DataSheet1.docx]

***Supplementary Material***

**Contents**

**Table S1.** Search strategy by database.

**Table S2.** Characteristics of the included studies.

**Table S3.** Quality assessment according to MINORS.

**Figure S1.** Funnel plot of studies that assessed the effectiveness between TNFi and non-TNFi.

**Figure S2.** Funnel plot of studies that assessed the effectiveness between Adalimumab, Etanercept, and Golimumab and Infliximab.

**Figure S3.** Effectiveness of (a) Adalimumab, (b) Etanercept, and (c) Golimumab compared to Infliximab.

**Figure S4.** Sensitivity analysis of Effectiveness of TNF inhibitors compared to non-TNF inhibitors.

**Figure S5.** Sensitivity analysis of Effectiveness of Adalimumab, Etanercept and Golimumab compared to Infliximab.

**Figure S6.** Sensitivity analysis of Effectiveness of biological disease-modifying anti-rheumatic drugs compared to Janus kinase inhibitors.

**Figure S7.** Sensitivity analysis of Effectiveness of biological disease-modifying anti-rheumatic drugs monotherapy compared to combination therapy.

**Figure S8.** Impact of varying degrees of unmeasured confounding bias on the proportion of studies with true risk ratio (RR): (A) < 0.9 (TNFi versus non-TNFi), (B) > 1.1 (Adalimumab, Etanercept, and Golimumab versus Infliximab), (C) < 0.9 (bDMARDs versus JAKi), and (D) < 0.9 (bDMARDs monotherapy versus combination therapy.

**Table S1.** Search strategy by database.

| **Database** | **Search terms** |
| --- | --- |
|  |  |
| Embase | 'rheumatoid arthritis'/exp AND ('adalimumab'/exp OR 'certolizumab pegol'/exp OR 'golimumab'/exp OR 'infliximab'/exp OR 'abatacept'/exp OR 'rituximab'/exp OR 'tocilizumab'/exp) AND ('biosimilar agent'/exp OR 'hydroxychloroquine'/exp OR 'methotrexate'/exp OR 'salazosulfapyridine'/exp) AND ('administrative personnel'/exp OR 'observational study'/exp OR 'cohort analysis'/exp) |
| Lilacs | tw:((rheumatoid arthritis AND (((((((((((((((adalimumab) OR certolizumab pegol) OR golimumab) OR infliximab) OR abatacept) OR rituximab) OR tocilizumab) OR antirheumatic agents)) AND ((methotrexate) OR hydroxychloroquine) OR sulfasalazine) OR biosimilar pharmaceuticals) OR biosimilar) OR biosimilars) OR biosimilarity) OR follow on biologics)) AND ((administrative personnel) OR (cohort studies) OR (cohort study) OR (studies, cohort) OR (study, cohort) OR (concurrent studies) OR (studies, concurrent) OR (concurrent study) OR (study, concurrent) OR (historical cohort studies) OR (studies, historical cohort) OR (cohort studies, historical) OR (cohort study, historical) OR (historical cohort study) OR (study, historical cohort) OR (analysis, cohort) OR (analysis, cohort) OR (cohort analyses) OR (cohort analysis) OR (closed cohort studies) OR (cohort studies, closed) OR (closed cohort study) OR (cohort study, closed) OR (study, closed cohort) OR (studies, closed cohort) OR (incidence studies) OR (incidence study) OR (studies, incidence) OR (study, incidence) OR (cohort studies) OR (cohort) OR (cohort analysis) OR (cohort study) OR (prospective cohort) OR (retrospective cohort) OR (retrospective cohort study) OR (prospective cohort study) OR (follow-up studies) OR (follow up studies) OR (follow-up study) OR (studies, follow-up) OR (study, follow-up) OR followup studies OR (followup study) OR (studies, followup) OR (study, followup) OR (epidemiologic studies OR retrospective studies OR longitudinal studies OR prospective studies))) AND ( db:("LILACS")) |
| Ovid | (rheumatoid arthritis) AND (adalimumab OR certolizumab pegol OR golimumab OR infliximab OR abatacept OR rituximab OR tocilizumab OR antirheumatic agents) AND (methotrexate OR hydroxychloroquine OR sulfasalazine OR biosimilar pharmaceuticals OR biosimilar* OR follow on biologics) AND (administrative personnel OR cohort Stud* OR concurrent stud* OR historical cohort stud* OR cohort anal* OR closed cohort stud* OR incidence stud* OR prospective cohort OR retrospective cohort OR retrospective cohort study OR prospective cohort study OR follow up stud* OR followup stud* OR follow-up stud* OR Epidemiologic Stud* OR retrospective stud* OR Longitudinal Stud* OR Prospective Stud*) NOT (review) |
| Pubmed | ("Arthritis, rheumatoid"[MeSH Terms] AND (((((((((((((((((("adalimumab"[MeSH Terms]) OR "certolizumab pegol" [MeSH Terms]) OR "golimumab"[Supplementary Concept] OR "golimumab"[All Fields]) OR "golimumab s"[All Fields]) OR "infliximab" [MeSH Terms]) OR "abatacept" [MeSH Terms]) OR "rituximab" [MeSH Terms]) OR "tocilizumab" [Supplementary Concept]) OR "tocilizumab" [All Fields]) OR "antirheumatic agents"[MeSH Terms])) AND (("methotrexate" [MeSH Terms]) OR "hydroxychloroquine" [MeSH Terms]) OR "sulfasalazine" [MeSH Terms]) OR "biosimilar pharmaceuticals"[MeSH Terms]) OR ("biosimilar"[All Fields] AND "pharmaceuticals"[All Fields]) OR "biosimilar pharmaceuticals"[All Fields]) OR "biosimilar"[All Fields]) OR "biosimilars"[All Fields]) OR "biosimilarity"[All Fields]) OR "follow on biologics"[All Fields])) AND (("administrative personnel"[MeSH Terms]) OR ("Cohort Studies"[Mesh]) OR (cohort study) OR (studies, cohort) OR (study, cohort) OR (concurrent studies) OR (studies, concurrent) OR (concurrent study) OR (study, concurrent) OR (historical cohort studies) OR (studies, historical cohort) OR (cohort studies, historical) OR (cohort study, historical) OR (historical cohort study) OR (study, historical cohort) OR (analysis, cohort) OR (analysis, cohort) OR (cohort analyses) OR (cohort analysis) OR (closed cohort studies) OR (cohort studies, closed) OR (closed cohort study) OR (cohort study, closed) OR (study, closed cohort) OR (studies, closed cohort) OR (incidence studies) OR (incidence study) OR (studies, incidence) OR (study, incidence) OR (cohort studies) OR (cohort) OR (cohort analysis) OR (cohort study) OR (prospective cohort) OR (retrospective cohort) OR (retrospective cohort study) OR (prospective cohort study) OR ("Follow-Up Studies"[Mesh]) OR (follow up studies) OR (follow-up study) OR (studies, follow-up) OR (study, follow-up) OR followup studies OR (followup study) OR (studies, followup) OR (study, followup) OR ("Epidemiologic Studies"[Mesh] OR "Retrospective Studies"[Mesh] OR "Longitudinal Studies"[Mesh] OR "Prospective Studies"[Mesh])) |
| Scopus | ALL(rheumatoid arthritis) AND ALL(adalimumab OR certolizumab pegol OR golimumab OR infliximab OR abatacept OR rituximab OR tocilizumab OR antirheumatic agents) AND ALL(methotrexate OR hydroxychloroquine OR sulfasalazine OR biosimilar pharmaceuticals OR biosimilar* OR follow on biologics) AND ALL(administrative personnel OR cohort Stud* OR concurrent stud* OR historical cohort stud* OR cohort anal* OR closed cohort stud* OR incidence stud* OR prospective cohort OR retrospective cohort OR retrospective cohort study OR prospective cohort study OR follow up stud* OR followup stud* OR follow-up stud* OR Epidemiologic Stud* OR retrospective stud* OR Longitudinal Stud* OR Prospective Stud*) |
| Web of Science | ALL=(rheumatoid arthritis) AND ALL=(adalimumab OR certolizumab pegol OR golimumab OR infliximab OR abatacept OR rituximab OR tocilizumab OR antirheumatic agents) AND ALL=(methotrexate OR hydroxychloroquine OR sulfasalazine OR biosimilar pharmaceuticals OR biosimilar* OR follow on biologics) AND ALL=(administrative personnel OR cohort Stud* OR concurrent stud* OR historical cohort stud* OR cohort anal* OR closed cohort stud* OR incidence stud* OR prospective cohort OR retrospective cohort OR retrospective cohort study OR prospective cohort study OR follow up stud* OR followup stud* OR follow-up stud* OR Epidemiologic Stud* OR retrospective stud* OR Longitudinal Stud* OR Prospective Stud*) |

**Table S2.** Characteristics of the included studies.

| **Study** | **Year** | **Study design** | **Secondary data sources** | **Treatment**  **(n)** | **Control**  **(n)** | **Age**  **(years)** | **Female**  **(%)** | **Adjustment** | **Inclusion criteria** |
| --- | --- | --- | --- | --- | --- | --- | --- | --- | --- |
| Acurcio | 2016 | Population-based cohort 2003-2010 | APAC/SIA  SIH  2010 Brazilian census | TNFi (14,313) | cDMARDs  (62,038) | TNFi: 49.0 (IQR 38.0–58.0)  cDMARDs: 52.0 (IQR 42.0–61.0)  Study population: 51.0 (IQR 41.0–60.0) | TNFi: 84.4  cDMARDs: 70.0  Study population: 81.7 | Age, sex, region of residence, income per capita, comorbidity score, general frailty, and calendar period of cohort entry | Patients ages ≥16 years;  ICD-10 codes M05.x, M06.x, and  M08.0;  Use of TNFi (infliximab,  etanercept, and adalimumab) or csDMARDs (azathioprine,  cyclosporine, chloroquine, hydroxychloroquine,  leflunomide, methotrexate, and sulfasalazine) |
| Bird | 2020 | Retrospective cohort  2015–2018 | Australian  OPAL dataset | Tofacitinib  (650) | bDMARD  (1,300) | Tofacitinib:  61.0 (SD 12.7)    bDMARD: 60.8 (SD 13.1) | Tofacitinib: 81.2  bDMARD: 81.2 | Age, sex, and treatment combinations | RA patients in the OPAL  dataset initiating treatment with  tofacitinib or a bDMARD (abatacept, adalimumab, anakinra, certolizumab pegol, etanercept,  golimumab, infliximab, rituximab, and tocilizumab) with at least 1 year of follow-up within the sample selection window |
| Chatzidionysio | 2014 | Population-based cohort 2005-2012 | Swedish Biologics Register (ARTIS) | Etanercept  (3,076)  Adalimumab  (1,802) | Infliximab (2,174) | Etanercept: 53.8 (SD 13.0)  Adalimumab: 54.4 (SD 13.4)  Infliximab: 52.7 (SD 15.0) | Etanercept: 77.0  Adalimumab: 77.2  Infliximab: 77.0 | - | RA patients within the ARTIS Register |
| Choi | 2021 | Population-based cohort 2014-2019 | Korean National Health Insurance  Service (HIRA) | Rituximab  (17)  Tofacitinib  (704)  Abatacept  (1,133) | Etanercept  (1,251)  Infliximab  (770)  Adalimumab  (1,658)  Golimumab  (1,133)  Tocilizumab  (1,352) | Etanercept: 53.9 (SD 13.7)  Infliximab: 55.7 (SD 12.3)  Adalimumab: 52.4 (SD 13.2)  Golimumab: 54.7 (SD 12.7)  Tocilizumab: 56.2 (SD 12.0)  Rituximab: 61.8 (SD 8.3)  Tofacitinib:  55.0 (SD 12.1)  Abatacept:  59.5 (SD 12.1) | Etanercept: 80.1  Infliximab: 82.5  Adalimumab: 80.5  Golimumab: 81.4  Tocilizumab: 81.3  Rituximab: 88.2  Tofacitinib:  81.8  Abatacept:  80.1 | Age, sex, hospital type, number of csDMARDs, Charlson comorbidity index, and enrollment year | Seropositive RA patients with at least first-line prescription of bDMARD or tofacitinib |
| Curtis | 2015 | Retrospective cohort  2007–2010 | Optum  Research Database | Abatacept  (295) | Adalimumab  (1,857)  Etanercept  (2,425)  Golimumab  (124)  Infliximab  (773) | Adalimumab: 48.5 (SD 9.6)  Etanercept: 48.2 (SD 10.1)  Golimumab: 48.1 (SD 10.3)  Abatacept: 49.7 (SD 9.4)  Infliximab: 49.6 (SD 9.4)  Study population: 48.6 (SD 9.8) | Adalimumab: 77.5  Etanercept: 77.6  Golimumab: 80.7  Abatacept: 83.7  Infliximab: 76.3  Study population: 77.8 | Age group, sex, geographic region, Charlson comorbidity score at index date, preindex DMARD use, and pre-index total health care cost | RA patients in Optum database receiving a biologic  approved for first-line treatment of moderate-to-severe RA  (abatacept, adalimumab, certolizumab pegol, etanercept, golimumab, or infliximab) |
| Curtis | 2021 | Prospective cohort  2016-2019 | AWARE Study | Golimumab-IV  (685) | Infliximab  (585) | Golimumab-IV: 60.9 (SD 13.4)  Infliximab: 58.0 (SD 12.9) | Golimumab-IV: 85.0  Infliximab: 79.5 | Age, sex, race, region, body mass index (BMI), weight, disease duration, CDAI, biologic-naïve, other medications, number of prior biologics received, prior TNFi therapy, selected  comorbidities, and smoking status | RA patients ≥ 18 years medically eligible for treatment with golimumab-IV or infliximab |
| Ebina | 2020 (a) | Prospective cohort  2001-2019 | Kansai Consortium for Well-being of Rheumatic  Disease Patients (ANSWER) | Tofacitinib  (101) | Abatacept  (273)  Adalimumab  (162)  Certolizumab pegol  (91)  Etanercept  (240)  Golimumab  (250)  Infliximab  (74)  Tocilizumab  (487) | Abatacept: 61.5 (SD 13.2)  Adalimumab: 55.4 (SD 14.8)  Certolizumab pegol: 54.1 (SD 15.4)  Etanercept: 55.5 (SD 15.7)  Golimumab: 60.5 (SD 14.6)  Infliximab: 53.5 (SD 12.6)  Tocilizumab: 58.1 (SD 14.1)  Tofacitinib: 59.7 (SD 13.6) | Abatacept: 81.3  Adalimumab: 87.7  Certolizumab pegol: 85.7  Etanercept: 82.1  Golimumab: 88.0  Infliximab: 79.5  Tocilizumab: 82.5  Tofacitinib: 77.2 | Age, sex, disease duration, concomitant PSL and MTX usage, and starting date of bDMARDs | RA patients according to the 1987 ACR or 2010 ACR/EULAR classification criteria |
| Ebina | 2020 (b) | Prospective cohort  2001-2019 | Kansai Consortium for Well-being of Rheumatic  Disease Patients (ANSWER) | JAKi  (24) | TNFi  (118)  Tocilizumab  (34)  Abatacept  (45) | - | - | Age, sex, disease  duration, concomitant PSL and MTX, treatment duration of Tocilizumab or Abatacept, and reasons of Tocilizumab or Abatacept discontinuation | RA patients according to the 1987 ACR or 2010 ACR/EULAR classification criteria primarily treated by abatacept or tocilizumab as first bDMARDs and then switched to either TNFi (infliximab, etanercept, adalimumab, certolizumab pegol, and golimumab – excluding biosimilar agents), abatacept, tocilizumab, or JAKi (tofacitinib or  baricitinib) |
| Gharaibeh | 2020 | Retrospective cohort  2012–2016 | IBM MarketScan  Commercial Claims and Encounters Database | Tofacitinib  (889) | Abatacept  (1,250)  Adalimumab  (4,986)  Certolizumab pegol  (387)  Etanercept  (5,266)  Golimumab  (577)  Infliximab  (969)  Tocilizumab  (451) | Abatacept: 50.3 (SD 9.1)  Adalimumab:  49.1 (SD 9.7)  Certolizumab pegol: 48.9 (SD 10.2)  Etanercept: 49.1 (SD 10.1)  Golimumab:  49.5 (SD 9.6)  Infliximab: 50.9 (SD 9.6)  Tocilizumab: 49.5 (SD 9.9)  Tofacitinib: 51.7 (SD 8.5)  Study population: 49.5 (SD 9.8) | Abatacept: 85.4  Adalimumab: 77.6  Certolizumab pegol: 84.8  Etanercept: 79.6  Golimumab: 81.6  Infliximab: 79.4  Tocilizumab: 84.9  Tofacitinib: 82.7  Study population: 80.0 | - | RA patients according to ICD-9 and 10 with an index claim for a target immunomodulator and continuous enrollment for 6 months before the index  date through 12 months after the index date |
| Harrold | 2015 | Prospective cohort  2000-2011 | Consortium of Rheumatology Researchers of North America Registry  (CORRONA) | Abatacept  (440) | TNFi  (958) | Abatacept:  57.7 (SD 12.4)  TNFi: 55.6 (SD 12.5) | Abatacept: 82.7  TNFi: 80.0 | Age, number of prior anti-TNF agents (one prior vs two  or more priors), duration of RA, swollen joint count, patient’s  global assessment, physician’s global assessment, and insurance  type | RA patients in CORRONA dataset with exposure to one or more TNFi but no prior use of non-TNFi |
| Kihara | 2017 | Population-based cohort 2010-2015 | The British Society for Rheumatology Biologics  Register for RA  (BSRBR-RA) | Tocilizumab  (217) | TNFi  (2,419) | TNFi: 57.0 (IQR 48.0–66.0)  Tocilizumab:  58.0 (IQR 49.0–66.0) | Tocilizumab: 76.0  TNFi: 73.0 | Age and sex | Bio-naive or bio-experienced RA patients ≥ 16 years  starting tocilizumab or TNFi therapy (etanercept, infliximab, adalimumab, or certolizumab pegol) |
| Lauper | 2018 | Prospective cohort  2009-2017 | Tocilizumab Collaboration  of European Registries in RA  (TOCERRA) | Tocilizumab monotherapy (771)  Tocilizumab combination therapy  (1773) | TNFi monotherapy  (1,404)  TNFi combination therapy  (4,660) | Tocilizumab monotherapy: 55.8 (IQR 47.5–64.5)  Tocilizumab combination therapy: 55.4 (IQR 46.8–62.2)  TNFi monotherapy: 54.5 (IQR 45.3–63.8)  TNFi combination therapy: 54.3 (IQR 44.0–61.9) | Tocilizumab monotherapy: 82.9%  Tocilizumab combination therapy: 80.2%  TNFi monotherapy: 83.2%  TNFi combination therapy: 79.9% | Sex, age, disease duration,  number of previously used bDMARDs, presence of rheumatoid factor (RF) or anticyclic citrullinated peptide antibodies, glucocorticoid use and daily dosage, functional disability (HAQ), DAS28-ESR, year of treatment initiation, and country of registry | RA patients ≥ 18 years, having used at least one bDMARD, baseline information on prior use of bDMARDs or csDMARDs and information on concomitant use of csDMARDs |
| Li | 2021 | Population-based cohort 2012-2017 | Taiwan National Healthcare Insurance Claims (NHIRD) | Tofacitinib  (787) | Etanercept  (2,341)  Adalimumab  (2,274)  Golimumab  (1,447)  Tocilizumab  (865)  Abatacept  (949) | Etanercept: 54.3 (SD 13.7)  Adalimumab: 53.8 (SD 13.6)  Golimumab: 55.0 (SD 13.0)  Tocilizumab: 56.1 (SD 13.6) Abatacept: 57.9 (SD 13.3)  Tofacitinib:  56.8 (SD 13.3)  Study population: 55.1 (SD 13.5) | Etanercept: 76.4  Adalimumab: 77.2  Golimumab: 79.1  Tocilizumab: 79.1  Abatacept: 80.8  Tofacitinib: 81.3  Study population: 78.3 | Index year, age, gender, Charlson Comorbidity Index, comorbidities, and use of concomitant medications | RA patients ≥ 18 years (ICD-9 codes 714.0 or ICD-10-CM codes M05.7–M05.9, M06.0,  M06.2, M06.3, M06.8, M06.9) receiving initial treatment of bDMARDs or tofacitinib |
| Neovius | 2015 | Population-based cohort 2003-2011 | Swedish Biologics Register (ARTIS) | Etanercept  (3,892)  Adalimumab  (2,349) | Infliximab (2,898) | Etanercept: 55.1 (SD 13.7)  Adalimumab: 55.7 (SD 13.3)  Infliximab: 56.7 (SD 13.1) | Etanercept: 77.0  Adalimumab: 75.0  Infliximab: 74.0 | Age, sex, period, education level, baseline HAQ, disease duration, concomitant DMARD, and general frailty | RA patients ≥16 years initiating their first TNFi therapy |
| Østergaard | 2007 | Population-based cohort  2002 | The Danish Database for Biological Therapies in Rheumatology (DANBIO) | Etanercept (22) | Infliximab  (278) | 56.0 (IQR 45–64) | 69.0 | - | RA patients in DANBIO database |
| Pappas | 2021 (a) | Prospective cohort  2001-2017 | Consortium of Rheumatology Researchers of North America Registry  (CORRONA) | bDMARD-naïve  Etanercept  (146)  bDMARD-experienced  Etanercept  (36) | bDMARD-naïve  Other TNFi  (274)  bDMARD- experienced  Other TNFi  (161) | bDMARD-naïve  Etanercept: 55.5 (SD 13.0)  Other TNFi: 58.6 (SD 13.7)  bDMARD-experienced  Etanercept: 54.8 (SD 12.4)  Other TNFi: 57.6 (SD 13.1) | bDMARD-naïve  Etanercept: 72.6  Other TNFi: 71.1  bDMARD-experienced  Etanercept: 83.3  Other TNFi: 72.7 | - | Adults ≥ 18 years old with a physician-confirmed diagnosis of RA who had achieved remission or LDA on combination therapy with TNFi and csDMARD and then discontinued the csDMARD |
| Pappas | 2021 (b) | Prospective cohort  2001-2018 | Consortium of Rheumatology Researchers of North America Registry  (CORRONA) | TNFi (4186) | Non-TNFi  (630) | TNFi: 56.9 (SD 12.7)  Non-TNFi: 62.7 (SD 13.0) | TNFi: 76.5  Non-TNFi: 79.8 | Gender, age, race, education, smoking status, body mass  index (BMI), median systolic blood pressure, history of hypertension, history of diabetes, history of anaemia, work status, private insurance, prior csDMARD use, median duration of RA, median tender joint count, median swollen joint count, median physician global assessment, and median patient global assessment | Patients aged ≥18 years with a documented diagnosis of RA, a valid CDAI score > 2.8, and no prior bDMARD or tsDMARD |
| Rahman | 2020 | Prospective cohort  2002-2017 | Biologic Treatment Registry Across Canada (BioTRAC) | Golimumab  (530)  Golimumab-IV  (157) | Infliximab  (890) | Golimumab: 57.7 (SD 13.0)  Golimumab-IV: 56.3 (SD 12.3)  Infliximab: 55.8 (SD 13.5) | Golimumab: 76.2  Golimumab-IV: 77.0  Infliximab: 86.8 | - | Bio-naive or  with ≤1 prior biologic agent exposure RA patients |
| Silvagni | 2018 | Retrospective cohort  2004-2013 | RECORD study by the Italian Society for Rheumatology | bDMARD  (799) | bDMARD + cDMARD  (3,679) | Study population: 55.48 (SD 12.69)  bDMARD: 54.90 (SD 12.97)  bDMARD + cDMARD: 55.61 (SD 12.62) | Study population: 77.53  bDMARD: 76.85  bDMARD + cDMARD: 77.68 | Sex, age, disease duration, Charlson Comorbidity Index (CCI), concomitant use of NSAIDs, GCs average dosage and previous bacterial infections | RA patients (copayment  exemption code 006.714.0) with at least one delivery of first-line approved bDMARDs (abatacept, adalimumab, certolizumab, etanercept, golimumab, infliximab, and tocilizumab) |
| Youssef | 2020 | Retrospective cohort  2010–2017 | Australian  OPAL dataset | Tofacitinib  (518) | Rituximab  (230)  Tocilizumab  (555)  Abatacept  (609)  Adalimumab  (1,788)  Certolizumab pegol  (1,868)  Etanercept  (832)  Golimumab  (57)  Infliximab  (457) | Rituximab:  68.0 (34.0-89.0)  Tocilizumab:  62.0 (19.0-93.0)  Tofacitinib:  62.0 (18.0-95.0)  Abatacept:  60.0 (18.0-96.0)  Adalimumab:  59.0 (18.0-93.0)  Certolizumab pegol:  60.0 (20.0-95.0)  Etanercept:  61.0 (19.0-96.0)  Golimumab:  61.0 (19.0-91.0)  Infliximab:  60.0 (26.0-85.0)  Study population: 61.0 (18.0-96.0) | Rituximab: 67.0  Tocilizumab: 76.0  Tofacitinib: 74.0  Abatacept: 77.0  Adalimumab: 75.0  Certolizumab pegol: 75.0  Etanercept: 75.0  Golimumab: 75.0  Infliximab: 74.0  Study population: 75.0 | - | RA patients ≥ 18 years registered in the OPAL dataset  starting a b/tsDMARD |
| Yun | 2015 | Retrospective cohort  2006–2010 | Medicare | Abatacept (2,129) | Adalimumab  (2,944)  Etanercept  (3,517)  Infliximab  (5,654) | Abatacept: 69.9 (SD 11.2)  Adalimumab: 63.0 (SD 12.9)  Etanercept: 63.7 (SD 13.0)  Infliximab: 69.3 (SD 10.1) | Abatacept: 83.5  Adalimumab: 83.0  Etanercept: 82.5  Infliximab: 79.8 | Gender, race, region, age, socio-economic status, nursing home resident, RA related conditions, outpatient infections, use of narcotics, antibiotics and RA medications, use of health services, other comorbidities, and calendar year of biologic initiation | ICD9 codes 714.x from rheumatologist visits,  followed by a prescription for, or an infusion of, methotrexate,  leflunomide, sulfasalazine, hydroxychloroquine,  an anti-TNF or non-anti TNF biologic approved for RA |

ACR: American College of Rheumatology; CDAI: Clinical Disease Activity Index; ICD: International Classification of Diseases; NR: Not reported.

**Table S3.** Quality assessment according to MINORS.

| **Study** | **Q1** | **Q2** | **Q3** | **Q4** | **Q5** | **Q6** | **Q7** | **Q8** | **Q9** | **Q10** | **Q11** | **Q12** | **Total** | **Methodological quality** |
| --- | --- | --- | --- | --- | --- | --- | --- | --- | --- | --- | --- | --- | --- | --- |
| Acurcio et al. (2016) | 2 | 2 | 2 | 2 | 2 | 2 | 0 | 0 | 2 | 2 | 2 | 2 | 20 | High |
| Bird et al. (2020) | 2 | 2 | 2 | 2 | 2 | 2 | 2 | 2 | 2 | 2 | 2 | 2 | 24 | High |
| Chatzidionysiou et al. (2015) | 2 | 2 | 2 | 2 | 2 | 2 | 0 | 0 | 2 | 2 | 2 | 2 | 20 | High |
| Choi et al. (2021) | 2 | 2 | 2 | 2 | 2 | 2 | 2 | 0 | 2 | 2 | 2 | 2 | 22 | High |
| Curtis et al. (2015) | 2 | 2 | 2 | 2 | 2 | 2 | 0 | 0 | 2 | 2 | 2 | 2 | 20 | High |
| Curtis et al. (2021) | 0 | 2 | 2 | 2 | 2 | 2 | 2 | 0 | 2 | 2 | 2 | 2 | 20 | High |
| Ebina et al. (2020a) | 2 | 2 | 2 | 2 | 2 | 2 | 2 | 0 | 2 | 2 | 2 | 2 | 22 | High |
| Ebina et al. (2020b) | 2 | 2 | 1 | 2 | 2 | 2 | 2 | 0 | 2 | 2 | 2 | 2 | 21 | High |
| Gharaibeh et al. (2020) | 2 | 2 | 1 | 2 | 2 | 2 | 2 | 0 | 2 | 2 | 2 | 0 | 19 | High |
| Harrold et al. (2015) | 2 | 2 | 2 | 2 | 2 | 2 | 1 | 0 | 2 | 2 | 2 | 2 | 21 | High |
| Kihara et al. (2017) | 2 | 2 | 2 | 2 | 2 | 2 | 2 | 0 | 2 | 2 | 2 | 2 | 22 | High |
| Lauper et al. (2018) | 2 | 2 | 2 | 2 | 2 | 2 | 0 | 0 | 2 | 2 | 2 | 2 | 20 | High |
| Li et al. (2021) | 2 | 2 | 2 | 2 | 2 | 2 | 2 | 0 | 2 | 2 | 2 | 2 | 22 | High |
| Neovius et al. (2015) | 2 | 2 | 2 | 2 | 2 | 2 | 0 | 0 | 2 | 2 | 2 | 2 | 20 | High |
| Ostergaard et al. (2007) | 2 | 0 | 2 | 2 | 0 | 0 | 0 | 0 | 2 | 2 | 2 | 2 | 14 | Moderate |
| Pappas et al. (2021a) | 2 | 2 | 2 | 2 | 2 | 2 | 2 | 0 | 2 | 2 | 2 | 2 | 22 | High |
| Pappas et al. (2021b) | 2 | 2 | 2 | 2 | 2 | 2 | 1 | 0 | 2 | 2 | 2 | 2 | 21 | High |
| Rahman et al. (2020) | 1 | 2 | 2 | 2 | 2 | 2 | 2 | 0 | 2 | 2 | 2 | 2 | 21 | High |
| Silvagni et al. (2018) | 2 | 2 | 1 | 2 | 2 | 2 | 2 | 0 | 2 | 2 | 2 | 2 | 21 | High |
| Youssef et al. (2020) | 2 | 2 | 1 | 2 | 2 | 2 | 2 | 0 | 2 | 2 | 2 | 2 | 21 | High |
| Yun et al. (2015) | 2 | 2 | 1 | 2 | 2 | 2 | 2 | 0 | 2 | 2 | 2 | 2 | 21 | High |

Items are scored as follows: not reported (0); reported but inadequate (1); reported and adequate (2).


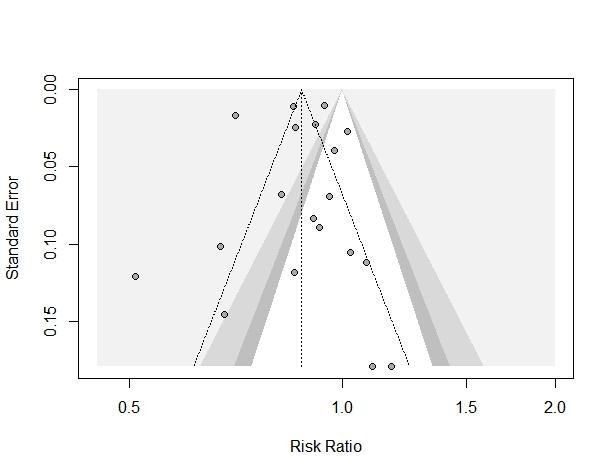
 **Figure S1.** Funnel plot of studies that assessed the effectiveness between TNFi and non-TNFi.


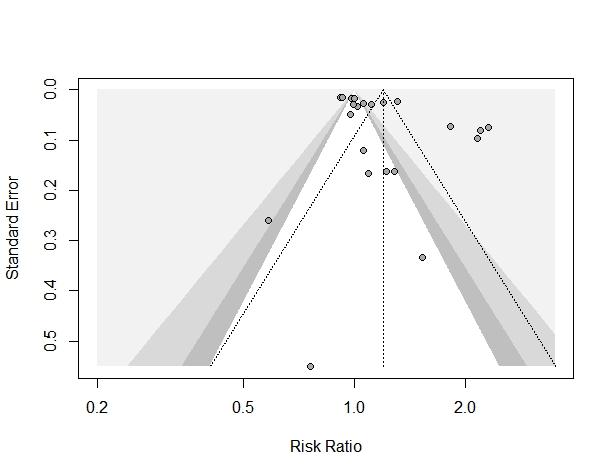
**Figure S2.** Funnel plot of studies that assessed the effectiveness between TNFi and TNFi (infliximab).


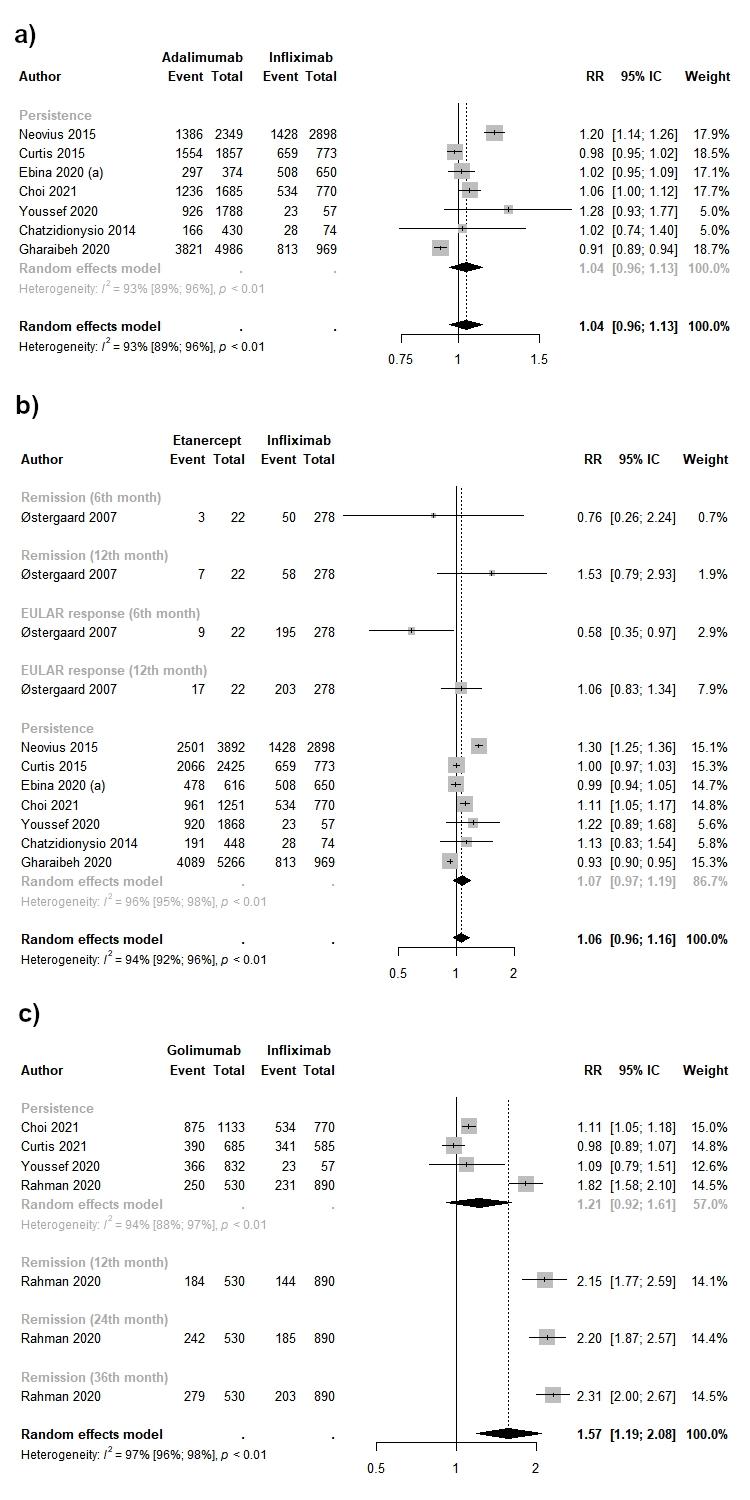


**Figure S3.** Effectiveness of (a) Adalimumab, (b) Etanercept, and (c) Golimumab compared to Infliximab.


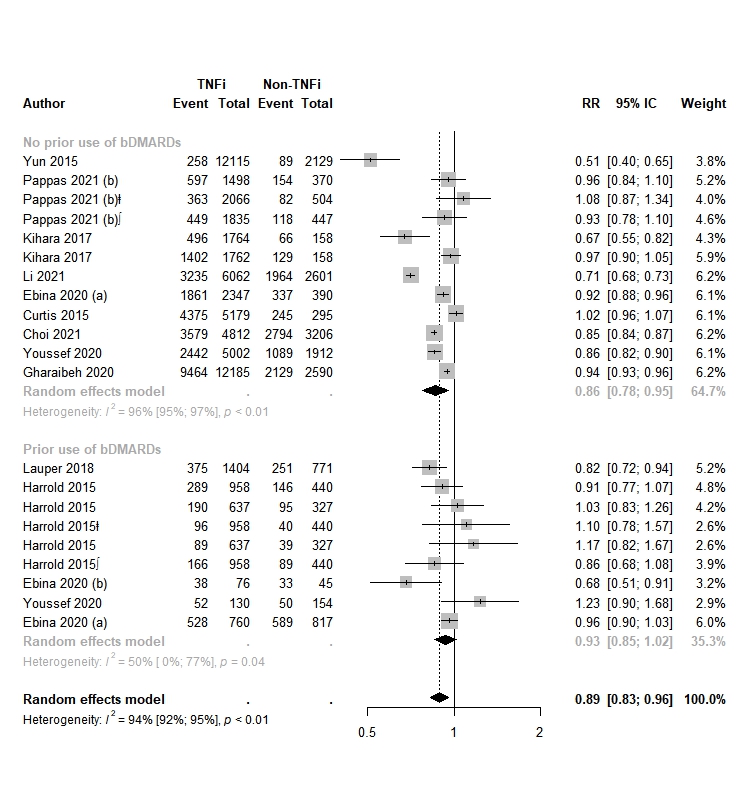


**Figure S4.** Sensitivity analysis of Effectiveness of TNF inhibitors compared to non-TNF inhibitors.

TNFi: TNF inhibitors; non-TNFi: non-TNF inhibitors; ⱡ: Remission based in CDAI; ∫: Remission based in DAS28.


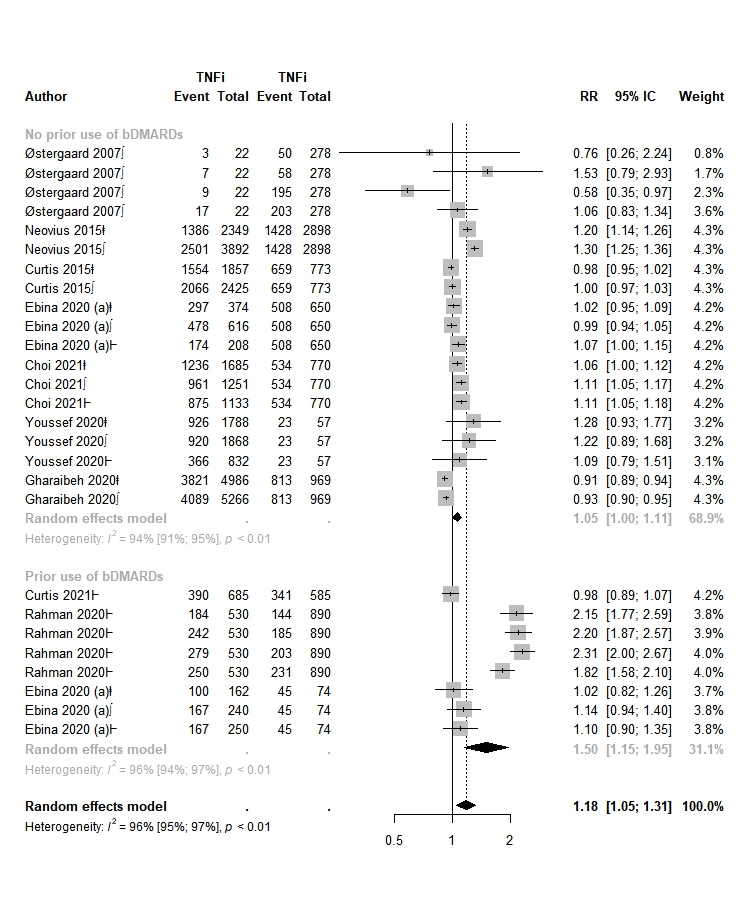
**Figure S5.** Sensitivity analysis of Effectiveness of Adalimumab, Etanercept and Golimumab compared to Infliximab.

TNFi: TNF inhibitors; ⱡ: Adalimumab; ∫: Etanercept; Ⱶ: Golimumab.


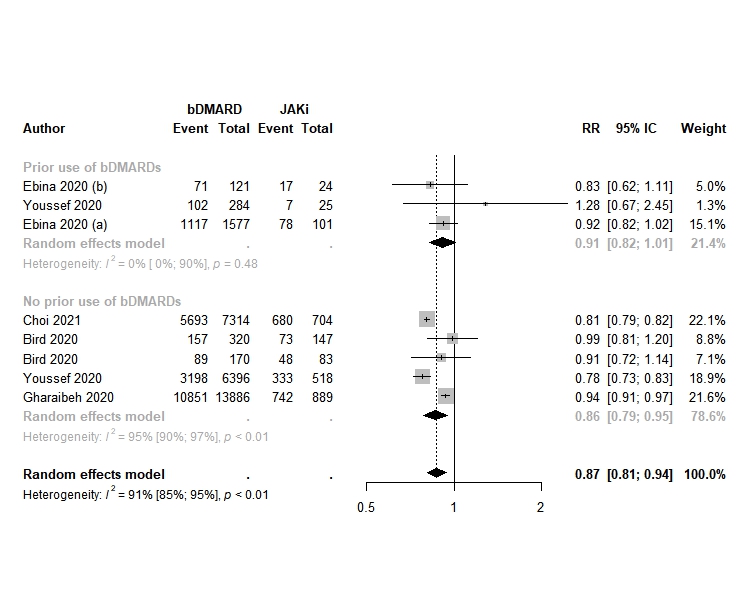
**Figure S6.** Sensitivity analysis of Effectiveness of biological disease-modifying anti-rheumatic drugs compared to Janus kinase inhibitors.

bDMARD: biological disease-modifying anti-rheumatic drugs; JAKi: Janus kinase inhibitors.


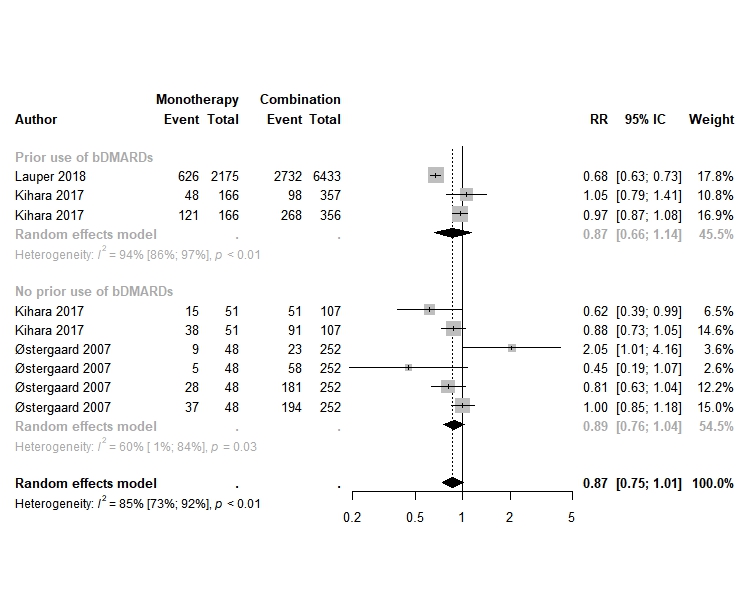
**Figure S7.** Sensitivity analysis of Effectiveness of biological disease-modifying anti-rheumatic drugs monotherapy compared to combination therapy.

Monotherapy: biological disease-modifying anti-rheumatic drugs monotherapy; Combination: biological disease-modifying anti-rheumatic drugs + Methotrexate.


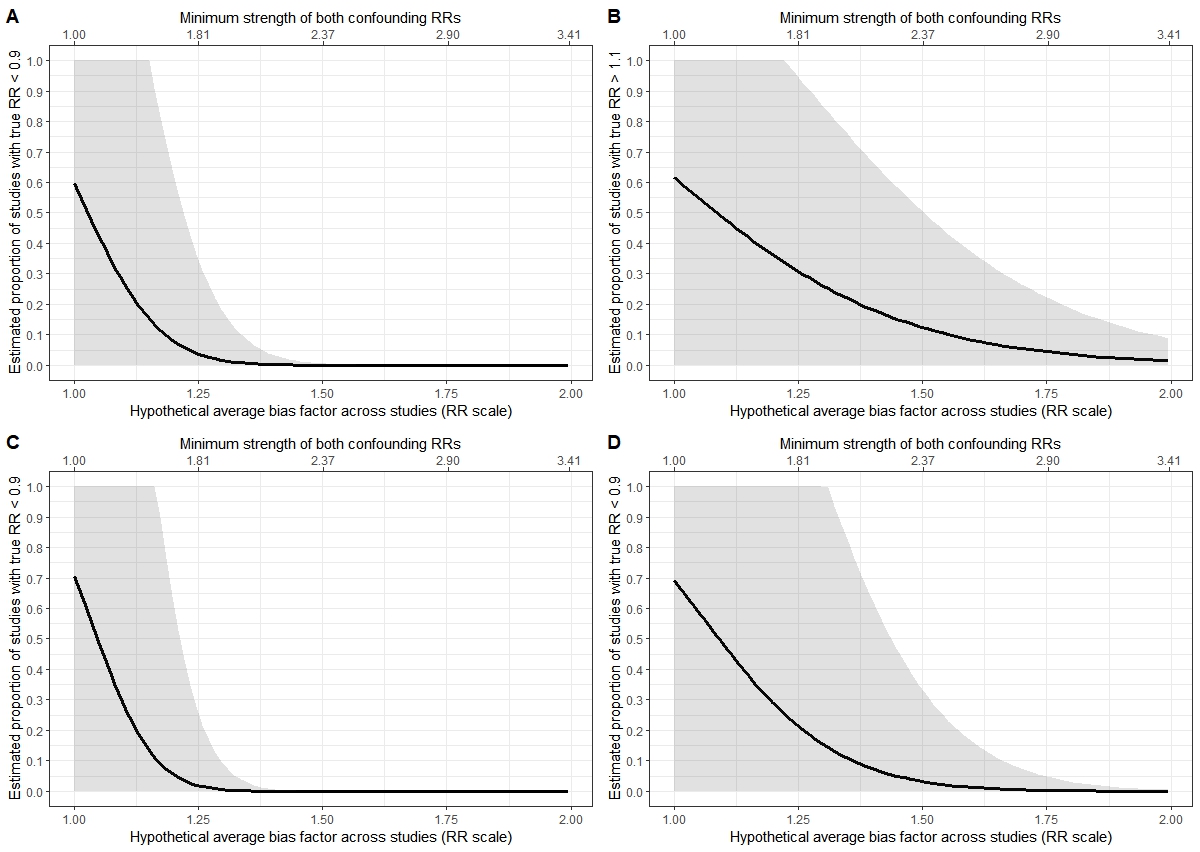


**Figure S8.** Impact of varying degrees of unmeasured confounding bias on the proportion of studies with true risk ratio (RR): (A) < 0.9 (TNFi versus non-TNFi), (B) > 1.1 (Adalimumab, Etanercept, and Golimumab versus Infliximab), (C) < 0.9 (bDMARDs versus JAKi), and (D) < 0.9 (bDMARDs monotherapy versus combination therapy.
